# Warming may offset impact of precipitation changes on riverine nitrogen loading

**DOI:** 10.1073/pnas.2220616120

**Published:** 2023-08-07

**Authors:** Gang Zhao, Julian Merder, Tristan C. Ballard, Anna M. Michalak

**Affiliations:** ^a^Department of Global Ecology, Carnegie Institution for Science, Stanford, CA 94305; ^b^Key Laboratory of Water Cycle and Related Land Surface Processes, Institute of Geographic Sciences and Natural Resources Research, Chinese Academy of Sciences, Beijing 100101, China; ^c^Department of Earth System Science, Stanford University, Stanford, CA 94305

**Keywords:** riverine nitrogen, global warming, precipitation, eutrophication, nutrient pollution

## Abstract

Climate change, especially in the form of precipitation and temperature changes, can alter the transformation and delivery of nitrogen on the land surface and to aquatic systems, impacting the trophic states of downstream water bodies. While the expected impacts of changes in precipitation have been explored, a quantitative understanding of the impact of temperature on nitrogen loading is lacking at landscape scales. Here, using several decades of nitrogen loading observations, we quantify how individual and combined future changes in precipitation and temperature will affect riverine nitrogen loading. We find that, contrary to recent decades, rising temperatures are likely to offset or even reverse previously reported impacts of future increases in total and extreme precipitation on nitrogen runoff across the majority of the contiguous United States. These findings highlight the multifaceted impacts of climate change on the global nitrogen cycle.

## Significance

Eutrophication threatens water resources across the globe. Earlier studies showed that climate-induced changes in precipitation would further exacerbate impacts, but observational records were not sufficiently long to conclusively pinpoint the role of future warming. Here, we use a long-term record of riverine nitrogen runoff to show that future warming will likely offset or even reverse the impact of precipitation changes, leading to a possible reduction in nitrogen runoff in the continental United States. Interestingly, future changes projected here are contrary to recent decades, when the impact of precipitation outweighed that of rising temperatures. Quantifying the impact of changes in climate on water quality outcomes at regional to continental scales is critical to ensuring water sustainability now and in the future.

Inland and coastal eutrophication has been widely observed across the globe (1–3) and can cause degradation of aquatic environments, with impacts such as cyanobacterial blooms (4), hypoxic “dead” zones (5), and greenhouse gas emissions (6, 7). Eutrophication is generally caused by an overenrichment of nutrients, primarily phosphorus and nitrogen, from both point and nonpoint sources. In particular, nitrogen (N) runoff from agricultural areas fuels biological productivity in many aquatic ecosystems, especially for coastal oceans (8). However, riverine N management is extremely challenging due to not only the large quantity of anthropogenic N that is added to the land surface (9, 10) but also the complexity of N transformation, the unknown composition of different forms of N and their transport in soils and inland water bodies. These delivery pathways are themselves impacted by changing climatic conditions, especially in the form of precipitation and temperature changes (11–16). How these alterations will translate to changes in N loading is of great concern for water quality protection and agricultural nutrient management.

Precipitation impacts water retention time, soil erosion (17), soil moisture (thus micro-anaerobic conditions) (18), and the duration of N-soil interactions (19), while temperature affects the biotic and abiotic transformation of N (20–22). Specifically, warming is likely to influence discharge (−) due to changes to evapotranspiration (+) (23) and alter the rates of nitrification (+), denitrification (+), anaerobic ammonium oxidation (+), mineralization (+), immobilization (±), leaching (±), biological fixation (−), and plant uptake (+) through direct (e.g., microbial activity) and indirect (e.g., dissolved oxygen and soil moisture) impacts (24, 25).

The impact of changes to total and extreme precipitation on nitrogen runoff has been documented (26), as has the potential substantial increase in N loading for the contiguous United States (CONUS) resulting from projected future increases in these two drivers (27). While these earlier studies were based on a limited observational record (1987 to 2007) that could only pinpoint the role of precipitation, a later study leveraged more recent observations to characterize the roles of both precipitation and temperature in impacting historical N loading trends across the CONUS over the period 1987 to 2012 (28). This study found that even though rising springtime temperatures tended to decrease N loading, this impact had been insufficient to offset precipitation-induced loading increases in the historical period.

How the tradeoff between precipitation and temperature changes will unfold under future climate conditions is unknown. One study based on the SPARROW model (29) examined this question using data from the CMIP3 (30) climate model intercomparison but only examined hydrologically average conditions and was not based on a landscape-scale empirical constraint of the sensitivity of nitrogen runoff to rising temperatures.

Here, we leverage an expanded multidecadal (1981 to 2017) record of riverine N loading in the CONUS that increases not only the duration of the observational period but also more than doubles the number of catchments relative to earlier work (28) to constrain the responses of N loading to climatic variability. A generalized additive model (GAM) (31) is used to explore the nonlinear relationship between the natural log of total annual riverine N loading and environmental covariates including climatic factors (precipitation and temperature), excess N input, land cover types, and the presence of tile drainage systems. For future periods (defined here as 2030 to 2059 for the mid-century and 2070 to 2099 for the late-century), we use the derived historical sensitivities to project the relative impacts of future precipitation and temperature changes on riverine N loading in the CONUS. Bias-corrected climate projections from 16 Coupled Model Intercomparison Project Phase 6 (CMIP6) models driven by three scenarios are used to represent a range of plausible future socioeconomic and emission scenarios.

## Results and Discussion

### Effects of Climate Variability on Nitrogen Loading.

**Fig. 1.**
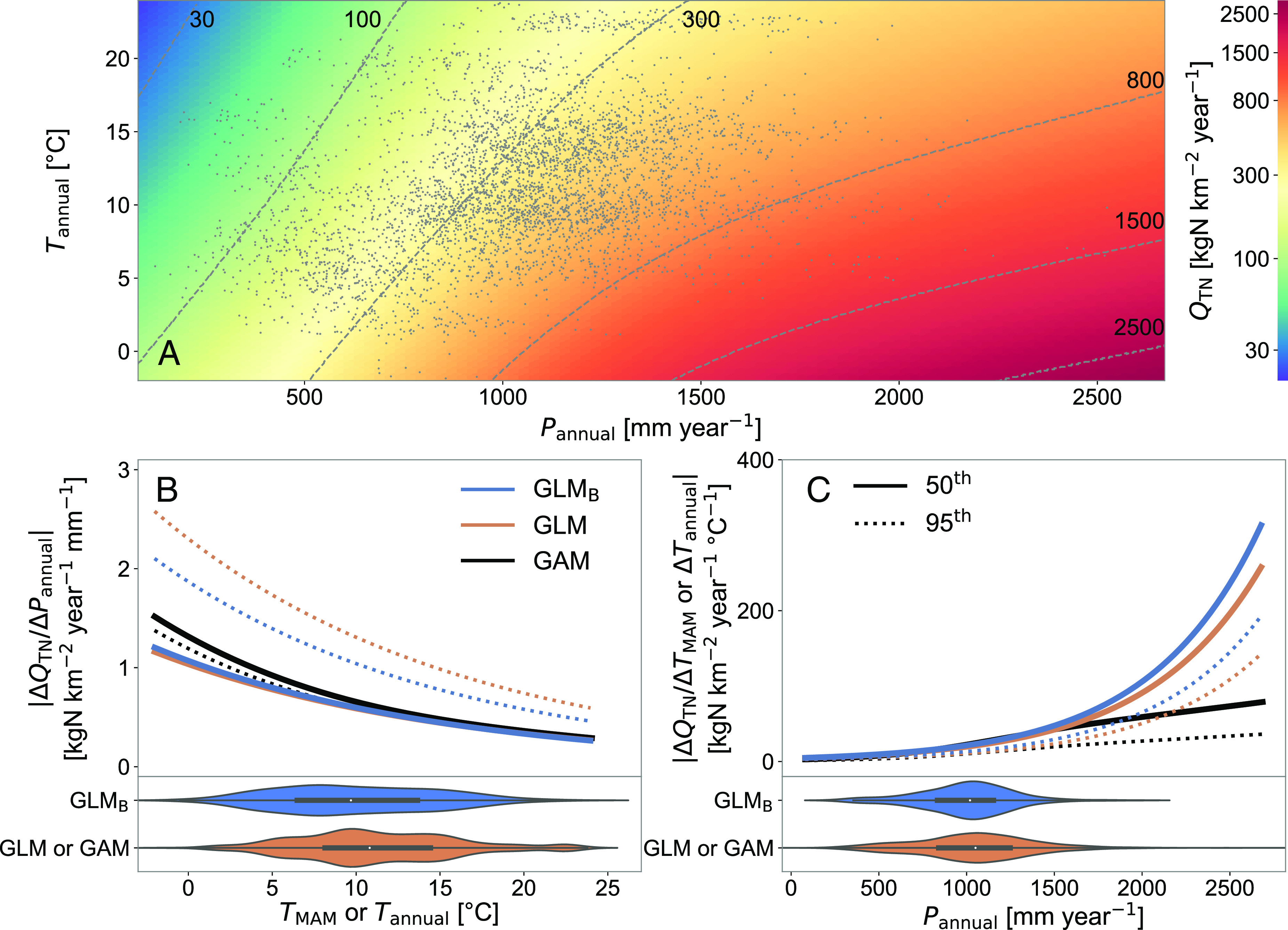
Response of annual nitrogen loading to changes in annual precipitation and temperature. (*A*) Joint effects of total annual precipitation ( $P_{\mathrm{annual}}$ ) and annual temperature ( $T_{\mathrm{annual}}$ ) on the annual nitrogen loading ( $Q_{\mathrm{TN}}$ ). (*B*) Sensitivity of $Q_{\mathrm{TN}}$ to annual (for the generalized linear model [GLM] and the generalized additive model [GAM], $T_{\mathrm{annual}}$ ) or March through May (for the generalized linear model of Ballard et al. ((28)) [GLM_B_], $T_{\mathrm{MAM}}$ ) temperature as a function of $P_{\mathrm{annual}}$ . (*C*) Sensitivity of $Q_{\mathrm{TN}}$ to $P_{\mathrm{annual}}$ as a function of $T_{\mathrm{annual}}$ (for GLM and GAM) or $T_{\mathrm{MAM}}$ (for GLM_B_). The violin plots represent the distribution of $P_{\mathrm{annual}}$ and $T_{\mathrm{annual}}$ (for GLM and GAM) or $T_{\mathrm{MAM}}$ (for GLM_B_) for the three models (GLM_B_, GLM, and GAM). Other covariates were held constant at historical median values. The sensitivities were calculated as absolute values of local slopes using 50th (solid lines) and 95th (dotted lines) percentile values of historical $T_{\mathrm{MAM}}$ or $T_{\mathrm{annual}}$ for panel *B* and 50th (solid lines) and 95th (dotted lines) percentile values of historical $P_{\mathrm{annual}}$ for panel (*C*).

We find that although increasing total precipitation ( $P_{annual}$ ) and extreme precipitation (*P*_*MAM*, *P* > 0.95_) lead to increased N loading, as expected, a saturation effect is observed at high precipitation values, wherein additional increases in precipitation have a reduced impact on increases in N loading (Fig. 1*A* and *SI Appendix*, Figs. S1*A*, S2, and S3). This effect is most pronounced when annual precipitation exceeds 1,500 mm and likely represents a limit to total available excess N in the soil column. Such conditions occur regularly in the Pacific Northwest and the Lower Mississippi River Basin, but can also occur during wet years in the Eastern United States. This effect is visible in Fig. 1*B*, where moving from the median annual precipitation to the 95th percentile of annual precipitation has little impact on the inferred sensitivity of loading to changes in precipitation when using the GAM model (solid line and dotted line, respectively). Conversely, using a linear model, whether based on the dataset used in Ballard et al. (28) (GLM_B_) or on the expanded dataset used here (GLM), yields an amplified impact of changes in precipitation on the sensitivity of loading (solid lines and dotted lines, respectively).

Increases in temperature ( $T_{annual}$ ), conversely, tend to decrease loading throughout the full temperature range when all other variables are held constant. Although warming alters a number of hydrological and biogeochemical processes that can either increase or decrease N loading (24, 25), the overall decrease suggests that the combined impact of those processes that reduce riverine nitrogen (e.g., denitrification, anaerobic ammonium oxidation, and immobilization) outweighs the combined impact of the mechanisms that push in the other direction (e.g., nitrification, mineralization, and leaching).

On average, across the range of precipitation and temperatures observed in the historical dataset, there is a 0.17% increase in loading for each millimeter increase in total annual precipitation (ranging from 0.04 to 0.24% depending on the baseline precipitation), a 0.12% increase in loading per millimeter increase in springtime extreme precipitation (ranging from 0.10 to 0.12%), and a 6.4% decrease in loading for each degree of warming (ranging from 5.4 to 7.1% depending on the baseline temperature) (*Materials and Methods*).

The sensitivity of N loading to temperature also changes with annual precipitation (Fig. 1*C*) and extreme precipitation, and conversely, the sensitivity to precipitation is impacted by temperature (Fig. 1*B*). The sensitivity of N loading to changes in temperature is greater when precipitation is higher, while the sensitivity to changes in precipitation decreases as temperature increases, implying a larger impact of climate variability on N loading in wet and cold regions (e.g., the Midwest).

Together, these results suggest that the impact of changes in precipitation will decrease as both precipitation and temperatures rise. This result implies that while Ballard et al. (28) found that the impact of historical increases in total and extreme precipitation throughout much of the CONUS on increases in N loading outweighed the impact of historical increases in temperature on decreases in N loading, the relative impact of these climatic factors may be different in the future as both temperatures and precipitation continue to rise over a substantial fraction of the CONUS.

### Future Changes in Nitrogen Loading.

We assess future N loading for watersheds in the CONUS using bias-corrected precipitation and temperature projections from 16 climate models participating in the Coupled Model Inter‐comparison Project phase 6 (CMIP6; see *Materials and Methods*). To isolate the impacts of climate change on N loading, we keep N surplus and land cover types at current values. Three Shared Socioeconomic Pathway (SSP) and emission scenarios were selected to illustrate a range of plausible future socioeconomic and radiative forcing conditions. These include the SSP126 “sustainability,” SSP245 “middle-of-the-road,” and SSP585 “fossil-fueled-development” scenarios (32). The major difference between these scenarios is caused by different greenhouse gas emissions trajectories under various climate mitigation strategies and global land cover land use changes. For each of these three scenarios, two future 30-y periods, namely the mid-century (2030 to 2059) and late-century (2070 to 2099) were used to characterize the long-term changes in N loading in the 21st century.

Both precipitation and temperature change substantially under the three scenarios (*SI Appendix*, Fig. S4). Generally, the greatest signal is seen under the fossil-fueled-development scenario, while the smallest signal is seen under the sustainability scenario. The divergence in scenarios is also more clearly seen by the late-century, with projections for the next couple of decades being relatively similar among the scenarios. The ensemble median of the 16 climate models projects an increase in both total annual precipitation and springtime extreme precipitation over most of the CONUS. Watersheds that are likely to have a drying trend in the future are primarily located in the Southwestern United States (*SI Appendix*, Fig. S4*D*). The warming magnitudes projected for the three scenarios range from an average of +1.8 °C to +5.0 °C over the CONUS relative to the period from 1988 to 2017. Despite these projected changes, the historical (1981 to 2017) precipitation and temperatures observed across the 258 catchments used to constrain the model developed here (*SI Appendix*, Fig. S5) encompass the vast majority of the range of precipitation and temperature values expected under future conditions (*SI Appendix*, Fig. S6). It is therefore reasonable to expect that the model developed here is able to represent expected responses under changing climate conditions.

**Fig. 2.**
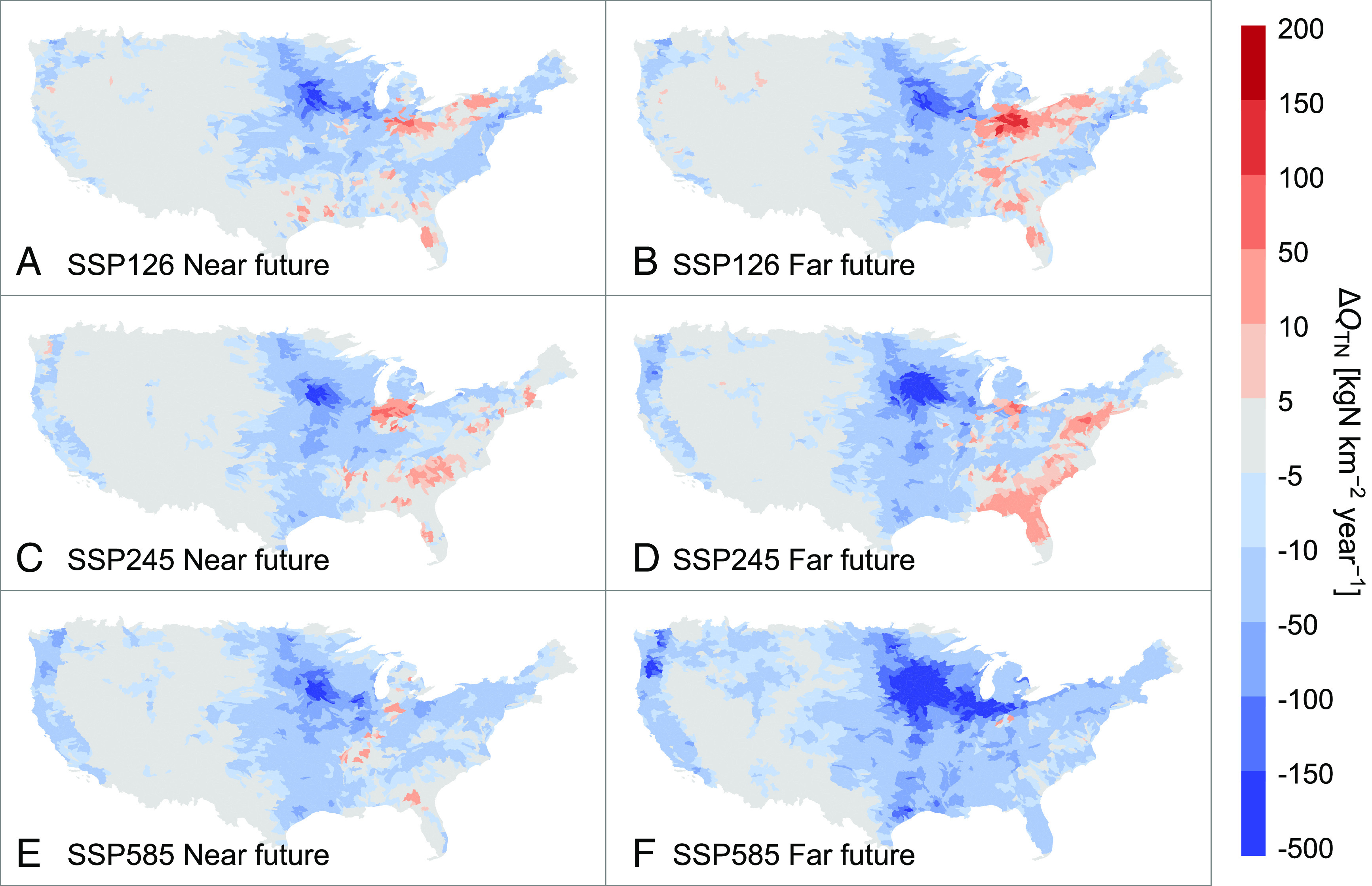
Projected changes in average annual nitrogen loading under different socioeconomic and emission scenarios. (*A* and *B*) show changes of future annual nitrogen loading ( $Q_{\mathrm{TN}}$ ) under the SSP126 “sustainability” for the mid-century (2030 to 2059) and the late-century (2070 to 2099). (*C* and *D*) are the same as (*A* and *B*) but for the SSP245 “middle-of-the-road” scenario. (*E* and *F*) are the same as (*A* and *B*) but for the SSP585 “fossil-fueled-development” scenario. The changes were calculated as the median value across the 16 climate models of the difference between the average value for a future period and the historical (1988 to 2017) average value.

All three scenarios result in an overall decrease in riverine N loading for the majority of the CONUS under current levels of N surplus (Fig. 2). This result suggests that the impact of increasing temperatures will outweigh the impact of changes in precipitation, which is opposite from what has been observed over the past 30 y (28). Taking the SSP245 middle-of-the-road scenario [a “likely” scenario given current policies (33)] as an example, the largest decrease in N loading is expected in the agricultural region in the upper Midwest, leading to a 4.6% overall reduction of N loading by mid-century and 7.0% by late-century for the Mississippi/Atchafalaya River Basin.

Although the majority of the CONUS is projected to see decreases in loading, some regions in the Southeast of the country and in the vicinity of the Great Lakes are projected to experience a further increase in N loading, with the impact of changes in precipitation outweighing the impact of projected warming. While the expected changes by mid-century are similar across the three scenarios, by late-century, the sustainability scenario would lead to more areas with increased N loading (Fig. 2*B* and *SI Appendix*, Fig. S4*D*), while under the fossil-fueled-development scenario, 74% of the CONUS is expected to experience a net reduction in riverine N due to massive warming (average +5.0 °C for CONUS; *SI Appendix*, Fig. S4*F*).

### Tradeoff between Precipitation and Temperature Effects on Nitrogen Loading.

**Fig. 3.**
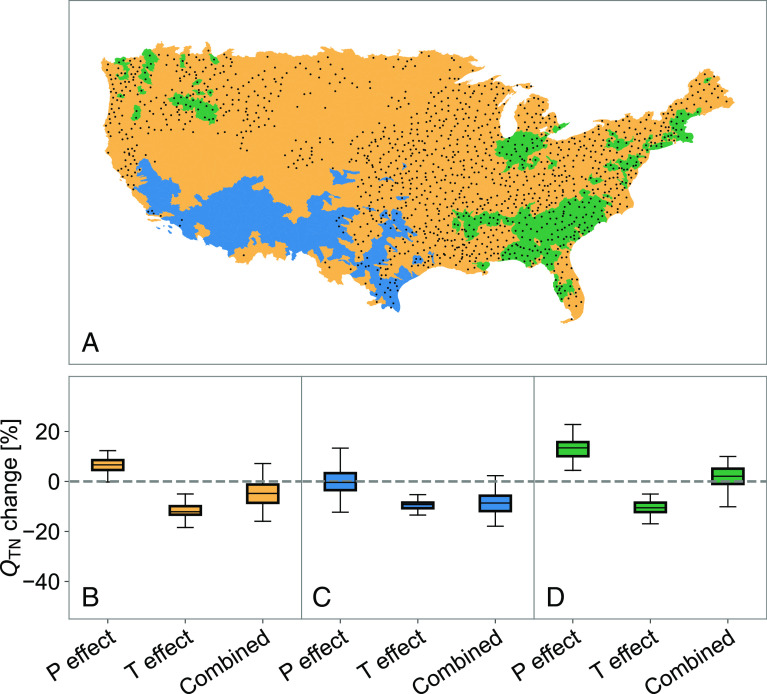
Tradeoff or compounding effects of precipitation and temperature changes on annual nitrogen loading. The mid-century under the SSP245 “middle-of-the-road” scenario was used as an example. (*A*) Different types of watersheds that are indicated in (*B*–*D*), with beige representing areas where changes in precipitation would lead to increases in loading but changes in temperature will likely more than offset this impact leading to a net reduction, blue representing areas where both temperature and precipitation changes are likely to lead to decreased loading, and green representing areas where the impact of changes in precipitation will likely outweigh that of changes in temperature leading to a net increase in loading. Stippling indicates areas where fewer than 80% of the climate models agree on the sign of net changes (34), and therefore where projections are less robust. The box plots in panel *B* (beige), *C* (green), and *D* (blue) show the net impact on the average loading ( $Q_{\mathrm{TN}}$ ) from each region, with the spread representing variability across the 16 climate models. “P effect” refers to the impact of changes in total annual precipitation and springtime extreme precipitation while “T effect” refers to the impact of changes in annual temperature and “Combined” refers to the combined impact thereof. The changes in expected median of $Q_{\mathrm{TN}}$ were calculated as the relative change between the mid-century (2020 to 2049) average value and the historical (1988 to 2017) average value.

Due to the opposing effects of increases in precipitation and temperature on N loading, future overall changes to riverine N will be the result of either the compounding impact of, or tradeoff between, these two climatic factors. While all regions across the CONUS are expected to experience warming, which will tend to decrease N loading, some regions will experience increases in annual and extreme precipitation that will more than offset the impact of warming and lead to an overall increase in loading (Fig. 3 *A* and *D*). For other regions, the effect of the increase in precipitation will be weaker than that of warming, and these regions will experience an overall decrease in loading (Fig. 3 *A* and *B*). A third subset of the CONUS will instead experience a decrease in precipitation, augmenting the decrease in N loading attributable to warming (Fig. 3 *A* and *C*).

Overall, the effect of warming will more than offset the effect of increasing precipitation for 76% of the area of the CONUS, taking the mid-century under the SSP245 middle-of-the-road scenario as an example (Fig. 3 *A* and *B*). This projection is most robust for the Western United States, where there is less variability across climate models in projections of precipitation (35). Continued increases in N loading are primarily expected in the Southeast, the Great Lakes region, and part of the Northeast and Northwest regions (Fig. 3 *A* and *D*), although there is considerable variability across climate models, making the projections for these regions less robust. Finally, for the Southwestern United States, the compounding effects of warming and drying lead to a robust projection of a net decrease in N loading (Fig. 3 *A* and *C* and *SI Appendix*, Fig. S4).

**Fig. 4.**
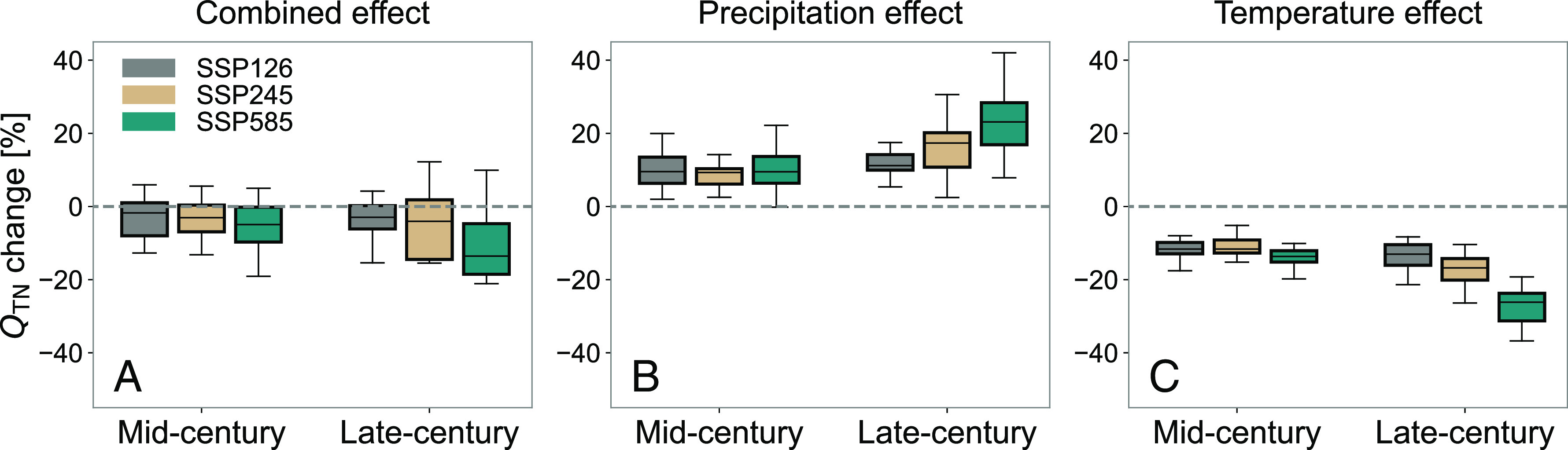
Changes in annual nitrogen loading for the CONUS in response to future precipitation and temperature changes. (*A*) Changes in $Q_{\mathrm{TN}}$ in response to the changes in total annual precipitation, springtime extreme precipitation, and annual temperature. (*B*) Changes in annual nitrogen loading ( $Q_{\mathrm{TN}}$ ) in response only to the changes in total annual precipitation and springtime extreme precipitation. (*C*) Changes in $Q_{\mathrm{TN}}$ in response only to the changes in annual temperature. The changes in $Q_{\mathrm{TN}}$ were calculated as the relative change between the mid- (2020 to 2049) and late-century (2070 to 2099) and the historical (1988 to 2017) periods for each climate model. The spread in the boxplots represents variability in projected change across the 16-model ensemble.

At the CONUS scale, the compound impact of changes in both precipitation and temperature leads to a net reduction in N loading (Fig. 4*A*), suggesting that the warming effect will be critical in regulating nitrogen loading in the future. Such an effect is especially notable by the late-century under the fossil-fueled-development scenario, which is associated with a faster warming rate. The median across the 16 climate models suggests reductions in N loading of 1.7 to 4.9% across the three scenarios for the mid-century, and 3.0 to 14% for the late-century.

The dominant role of warming is opposite to that observed in recent decades, when the impact of changes in precipitation outpaced that of warming for a net increase in N loading (28). An additional sensitivity analysis (*SI Appendix*, Fig. S7) demonstrates that the nonlinearity in the response to precipitation and temperature under the more intense warming expected in the future (see Fig. 1*A* and associated text) is the cause of the dominant role of temperature under future climate conditions. This sensitivity analysis was conducted by taking the linear model from Ballard et al. (28) (GLM_B_) and applying it with temperature and precipitation from the CMIP6 multi-model ensemble. This comparison highlights the value of the extended training dataset available here and the resulting inference of the nonlinear response to changes in climate, specifically the decreasing sensitivity of N loading to precipitation at high temperatures (Fig. 1).

Quantitatively, the impact of projected changes in precipitation for the CONUS is consistent with a past study (27), with projected increases in N loading across the three examined scenarios of 9.3 to 9.5% for the mid-century and 11 to 23% for the end of the century (Fig. 4*B*), expressed as a median across the 16 climate models. The low variability across scenarios in the mid-century reflects the similarity in projected changes to precipitation during this period (*SI Appendix*, Fig. S4*D*). For the last three decades of the century, however, the projected increase in total and extreme precipitation under the fossil-fueled-development scenario leads to a large (23%) increase in N loading, which is consistent with the findings of Sinha et al. (27), who reported a 19% increase for CONUS toward the end of the 21st century as a result of projected changes in precipitation alone.

The new constraint on the incremental role of temperature, however, suggests that the impact of changes in temperature on future reductions in N loading will be even greater than that of precipitation (Fig. 4*C*). This conclusion is robust to the modeling uncertainty of GAM (*SI Appendix*, Figs. S8 and S9; see *Materials and Methods*). Quantitatively, the increases in N loading under the middle-of-the-road scenario for the mid- and late-century are 9.3 and 17% as a result of changes in precipitation alone, while the decreases in N loading are 12 and 17% as a result of changes in temperature alone.

Critically, we find that climate model uncertainty in projections of temperature and precipitation contributes substantial uncertainty to future N loading estimates, highlighting the wide range of potential water quality conditions we may expect in the coming decades. Indeed, for both the mid- and late-century, the combined effect of temperature and precipitation leads to a net increase in N loading for about a quarter of climate models for all but one period–scenario combination (Fig. 4*A*). Also, the lower bounds of changes in annual nitrogen loading are similar for the mid- and late-century, ranging from −13 to −19% for the former and from −15 to −21% for the latter.

### Implications for N Loading under Climate Change.

Our analysis reveals the importance of temperature in regulating watershed-scale riverine N loading and reducing or reversing the impacts of increases in precipitation. Future projections based on all three socioeconomic and emission scenarios suggest a decrease in overall N loading, with the temperature impact outweighing that of precipitation. This projected trajectory in the future contrasts with the historical findings from Ballard et al. (28). In all cases, however, about a quarter of climate models project conditions under which N loading would continue to increase at the scale of the CONUS, which represents a major uncertainty for managing these complex systems.

Together, these findings have substantial implications for the global nitrogen cycle (9), coastal ecosystem health (36), and agricultural/aquacultural management (37). Given the regional nature of the tradeoff between, or compounding effects of, precipitation and temperature on N loading, tailored planning is needed within the context of societal decisions (38) to balance nitrogen in terrestrial and aquatic ecosystems.

The inferred decrease in loading in response to warming is likely primarily the result of higher denitrification rates through enhanced microbial activity (39, 40), reduced oxygen concentrations caused by increased respiration and reduced solubility (41, 42), decreases in discharge due to enhanced evapotranspiration (23), and other biotic/abiotic processes (24, 25). The effect of temperature on enhanced denitrification rates has been widely investigated in laboratory settings. Based on 47 such experiments gleaned from the literature, the median reported $Q_{10}$ value (i.e., the fractional change in the rate of a biochemical process for a 10 °C rise of temperature) is 2.7 (interquartile range of 2.1 to 3.7; *SI Appendix*, Table S1). Converting the temperature response inferred here based on watershed-scale monitoring to an equivalent $Q_{10}$ value leads to an estimate of 1.1 (1.05 to 1.23; see *Materials and Methods*), which is smaller than the lower end of the range inferred based on the laboratory-based studies. This is likely due to the fact that not all excess N will be channeled into the denitrification process (9, 43) and that our watershed-scale response includes temperature impacts through mechanisms other than denitrification (e.g., nitrification, mineralization, algal uptake, and burial). Thus, the overall impact of temperature, as observed here, may be smaller than the denitrification signal alone from laboratory-based experiments (24).

While warming tends to decrease N loading, it can exacerbate eutrophication via other downstream processes such as by intensifying stratification (44), favoring harmful algae and cyanobacteria (45), enhancing marine nitrogen fixation (46), and suppressing herbivorous zooplankton (47). In addition, land cover change or the expansion in the use of tile drainage can interact with climatic changes to further impact the transport and transformation of nitrogen. Thus, more mechanistic understanding across scales is needed to project eutrophication risks under a changing climate. Our study provides a critical baseline for evaluating the impacts of a changing climate on riverine nitrogen and also highlights the need for a holistic understanding of the interplay between the global nitrogen cycle and anthropogenic climate change.

## Materials and Methods

### Observed N Loading.

Observed annual N loads ( $Q_{\mathrm{TN}}$ ) were quantified using the Weighted Regressions on Time, Discharge and Season (WRTDS) approach (48, 49), which calculates continuous loading of constituents based on sampled concentration values and continuously monitored streamflow data.

Relative to previous studies (26, 28), we have expanded the spatiotemporal coverage of the data used for model development by an order of magnitude (*SI Appendix*, Fig. S5*A* and Table S2). We expanded the period of record to 1981 to 2017. With the longer record, we were able to expand the dataset to a total of 258 monitoring sites managed by the United States Geological Survey, selected based on data availability of observed streamflow (at least 20 y of continuous daily data) and available measurements of total nitrogen concentration (at least 100 measurements) (28).

For each site, the annual N loading was calculated for the years with at least six measurements to assure the representativeness of the annual data (48). In total, 4,270 annual $Q_{\mathrm{TN}}$ observations were assembled for the 258 sites from 1981 to 2017 (*SI Appendix*, Table S2), yielding an average of between 16 and 17 annual observations per catchment. In addition, previous studies (26, 28) relied on net anthropogenic nitrogen input (NANI) (50) to represent nitrogen inputs into the system, and NANI estimates are only available at five-year intervals. Here, we instead rely on a more recent dataset of annual nitrogen surplus data (10, 51) (as described in the following section), further increasing the data available for model development by a factor of five. Overall, the assembled dataset can better represent spatial heterogeneity, especially in the southwestern United States (*SI Appendix*, Fig. S5), interannual variability, and long-term trends, all of which are important for discerning the emerging impacts of warming and precipitation changes.

### Environmental Covariates.

The environmental covariates, representing the impacts of nitrogen input, climatic conditions, and land cover/land use on nitrogen loading, were assembled for each of the 4,270 annual $Q_{\mathrm{TN}}$ observations. First, the shapefiles of the contributing catchment for each of the 258 sites were produced by either collecting them from the Geospatial Attributes of Gages for Evaluating Streamflow Version II (GAGES-II) database (52) or delineating them using the 15-arcsecond flow direction map from the HydroSHEDS database (53). Second, the corresponding independent covariates for each of the 4270 catchment-year pairs were calculated by averaging the overlapping grids from the covariate maps.

Following Sinha and Michalak (26) and Ballard et al. (28), 52 candidate covariates were defined and calculated to represent the environmental impacts on N loading (*SI Appendix*, Table S3). Specifically, we used the N surplus (Eq. **1**) data from Byrnes et al. (10) to represent the excess N that was added to the land surface of the contributing catchments (*SI Appendix*, Fig. S5*A*).

[1] $$N_{surplus}=N_{dep}+N_{fert}+N_{fix}+N_{man}+N_{hum}-N_{crop}$$

where dep , fert , fix , man , hum , and crop are atmospheric deposition, inorganic fertilizer, biological fixation, manure, human waste, and crop/livestock uptake, respectively. N surplus is similar to the NANI data used by earlier works (26, 28) but has substantially extended temporal coverage (i.e., every year from 1930 to 2017), which enables the expansion of $Q_{\mathrm{TN}}$ observations that can be used for model development. Here, both N surplus and its log-transformed values, as well as their combination for the previous two years were considered as candidate covariates to represent both fast N transport at the surface of soil columns and slow N release from short-term legacy sources.

For climate covariates, we defined annual, seasonal, and extreme precipitation metrics, along with annual and seasonal temperature covariates (*SI Appendix*, Table S3). These covariates were calculated for each catchment-year pair based on the Precipitation-elevation Regressions on Independent Slopes Model (PRISM) dataset (54). Specifically, the extreme precipitation metrics were calculated for both annual and springtime (March to May) using total precipitation that exceeds a specific percentile value (i.e., 90th, 95th, and 99th) during the historical period from 1981 to 2010 (26, 28).

By including precipitation, temperature, and land use in our model, we also implicitly account for the major drivers of discharge while avoiding the need to model discharge explicitly. The effectiveness of this approach is illustrated in *SI Appendix*, Figs. S5, S11, and S12, where we show that by accounting for the factors illustrated in *SI Appendix*, Fig. S1, we are able to capture the vast majority of the spatial and temporal variability in nitrogen loading across and within a wide variety of geographic regions (*SI Appendix*, Fig. S5) and temperature and precipitation conditions (*SI Appendix*, Fig. S6).

The land use covariates were derived from the National Land Cover Database 2006 (55) to represent the average state of land use during the 37 y. Due to the minimal change in land use in the United States during the study period (*SI Appendix*, Fig. S10), these covariates were used as time-invariant values to reflect the spatial variability of nitrogen loading. Change in vegetation properties can affect nitrogen flux via uptake, but this effect is captured via other variables in the model, including atmospheric deposition (which enhances growth) (56), precipitation, and temperature (57). The major five land use types [i.e., developed (D), cultivated (C), forest (F), shrubland and herbaceous (SH), and wetlands (W)] and their combinations were all considered as candidate covariates. In addition, although overlapped with the cultivated land use, the areal percentage of tile drainage was included because such systems are generally linked to disproportionate N leaching (58, 59).

### Historical GAM Fitting.

A GAM (31) was used to characterize the nonlinear relationships between the natural log of $Q_{\mathrm{TN}}$ and the selected covariates using a Gaussian error distribution. This means that $Q_{\mathrm{TN}}$ is expected to follow a conditional lognormal distribution. The back transformation of a model prediction via the exponential function thus represents the median prediction of $Q_{\mathrm{TN}}$ . To select the best model, each candidate model was formulated by combining up to the maximum allowed numbers of candidate covariates from each category (*SI Appendix*, Table S3) (26, 28). For instance, at most one extreme precipitation metric can be selected while a maximum of four land use types can be selected. Model selection was conducted by minimizing the Bayesian Information Criterion (BIC) (60) for candidate models. A model is superior to another when the difference of BIC values is greater than two (28). Because BIC has a penalty term based on the number of covariates and their effective degrees of freedom, the model with the lowest BIC also limits the likelihood of overfitting.

The final selected model includes nine covariates (*SI Appendix*, Fig. S1), namely natural-log-transformed N surplus [ $ln(N_{\mathrm{surplus}})$ ], natural-log-transformed N surplus of the previous two years [ $ln(N_{surplus,-1\&-2})$ ], total annual precipitation ( $P_{\mathrm{annual}}$ ), total spring-time (March, April, and May) precipitation that exceeds the 95th percentile of historical (1981 to 2010) values ( $P_{MAM,p>0.95}$ ), average annual temperature ( $T_{\mathrm{annual}}$ ), percent coverage of developed land ( ${\mathrm{LU}}_{D}$ ), percent coverage of cultivated land ( ${\mathrm{LU}}_{C}$ ), percent coverage of forest and shrubland ( ${\mathrm{LU}}_{F,SH}$ ), and percent of land with tile drainage ( $L_{\mathrm{TD}}$ ). Only one other model had a BIC difference from the selected model of less than two. The only difference between these two models was the use of the total spring-time (March, April, and May) precipitation that exceeds the 90th percentile of historical (1981 to 2010) values ( $P_{MAM,p>0.90}$ ) in lieu of the 95th percentile ( $P_{MAM,p>0.95}$ ). Even among all the models with a BIC difference of less than twenty, the only differences relative to the selected model were minor substitutions in the specific extreme precipitation variable used and the use of springtime vs. annual temperature, further confirming the robustness of the primary drivers captured by the selected model.

The model is effective at capturing both spatial and temporal variability in nitrogen loading (*SI Appendix*, Figs. S5, S11, and S12). This is because it leverages observations spanning over three decades (see Observed N loading). The overall performance of the model is presented in *SI Appendix*, Fig. S5, which demonstrates that the model effectively reproduces the space and time variability in the calibration dataset. *SI Appendix*, Fig. S11 then bins catchments by region and shows that the model developed here does equally well at capturing variability (in both space and time) within individual regions as it does across the CONUS as a whole. Taking the analysis one step further, *SI Appendix*, Fig. S12 shows that the model can even reproduce temporal variability in the historical period for individual catchments.

The selected covariates and their impact on N loading are generally consistent with those of the generalized linear model (GLM) of Ballard et al. (28) (*SI Appendix*, Figs. S1 and S2), but with some notable additions and observed nonlinearities. The positive effect of total annual precipitation ( $P_{\mathrm{annual}}$ ) and the springtime extreme precipitation ( $P_{MAM,p>0.95}$ ) reflect a higher N yield with more precipitation. For temperature, a negative relationship is observed, representing the net effect of the various processes impacted by temperature (see *Results* and *Discussion*). The selected excess N terms are N surplus for the current year [ ${ln(N}_{\mathrm{surplus}})$ ] and for the previous two years [ $ln(N_{surplus,-1\&-2})$ ]. Specifically, the $ln(N_{surplus,-1\&-2})$ term represents a short-term N legacy effect and can be attributed to the N residual that was stored in the deeper soil and transported by slow-moving groundwater (61, 62). The land cover types ${\mathrm{LU}}_{D}$ , ${\mathrm{LU}}_{C}$ , and $L_{\mathrm{TD}}$ all represent disturbed or managed land, which leads to a higher anthropogenic N loading from sources such as urban wastewater, agricultural runoff, and fast drained agricultural interflow. In particular, the positive slope of the $L_{\mathrm{TD}}$ underscores the importance of N leaching in tile drainage systems. For ${\mathrm{LU}}_{F,SH}$ , the GAM result shows a convex relationship with a first increasing and then decreasing response. The relative importance of this variable is low and likely indicates a residual relationship that is not captured by other covariates.

To assess the potential impact of nested catchments among the training dataset, we also ran a sensitivity analysis where all fully nested catchments were removed, yielding 196 nonnested catchments. We then used data from these catchments to retrain GAM (*SI Appendix*, Fig. S13). The resulting model is very similar to the original, and especially so for the climate covariates (i.e., precipitation, extreme precipitation, and temperature). Thus, the fact that some catchments are nested does not impact the climatic sensitivities of nitrogen loading. Minor differences between the original model and the retrained model are the two nitrogen surplus terms [i.e., $ln(N_{\mathrm{surplus}})$ and $ln(N_{surplus,-1\&-2})$ ]. Because these variables are highly correlated, their combined impact is very similar across the two models.

### Future Climate Scenarios.

Climate data from the Phase 6 of the Coupled Model Intercomparison Project (CMIP6) were used for calculating the change of annual N loading in the future (63). Specifically, three alternative SSP scenarios and 16 General Circulation Models (GCMs) under each scenario (*SI Appendix*, Table S4) were selected to account for the projection uncertainties. SSP126 represents the mitigation scenario by coupling the “Sustainability” SSP with the Representative Concentration Pathway 2.6 (RCP2.6) radiative forcing trajectory. The SSP245 “middle-of-the-road” scenario represents a modest climate regulation strategy and is deemed as the “likely” scenario given current policies, while the SSP585 “fossil-fueled-development” scenario represents a high emission future and is used as the upper bound of future temperatures although such scenario is “highly unlikely” (33, 64). The selection of models was based on the data availability of daily precipitation and monthly temperature in the Scenario Model Intercomparison Project (ScenarioMIP) (63), which is one of the CMIP6-Endorsed MIPs that depicts how the Earth system evolves under future emissions and land use changes. In our study, GCMs with an earth system model (ESM) component were prioritized if a modeling group had multiple options (e.g., GFDL-CM4 vs. GFDL-ESM4) (*SI Appendix*, Table S4) to fully consider the interplay between carbon emissions with the Earth system.

For each scenario and each model, gridded daily precipitation and monthly temperature data for both the historical (1950 to 2014) and future periods (2015 to 2099) were collected from Google Cloud Storage. The time series of daily precipitation and monthly temperature for each 8-digit hydrologic unit (HUC8) watershed were then generated by averaging the grids that intersect with the extent of the watershed. HUC8 watersheds were used because their areas, total stream lengths, and in-stream travel times were comparable to those of the catchments used in the training dataset (*SI Appendix*, Table S5) and thus the signals of key nitrogen processes that are captured in our model will also be applicable for HUC8 watersheds throughout the CONUS. For each of the watersheds, the systematic biases inherited from the GCM were corrected using scaled distribution mapping (SDM) (65). Similar to quantile delta mapping, SDM calculates the biases for each quantile of the historical distribution and thus is appropriate for applications that consider both average state and extreme values of precipitation. Compared to other methods that are based on quantile mapping, SDM can better preserve the changes projected by GCMs (65). Also, although there are differences among bias-correction methods, the uncertainty resulting from the choice of a particular bias-correction method is considerably smaller relative to the uncertainty resulting from differences between emission scenarios and climate models (66).

The bias-correction step was implemented using a reference period from 1981 to 2010 and the corresponding observations were based on the PRISM dataset (54) for each watershed. The biases were then calculated by comparing the cumulative distribution between the observations and climate model results for the reference period. Specifically, biases in daily precipitation and monthly temperature were calculated as multiplicative and additive, respectively (65). These biases were then corrected for both the historical (1950 to 2014) and future (2015 to 2099) periods for each watershed, model, and scenario.

The results shown in (Figs. 3 and 4) include uncertainty based on the spread across the 16-model ensemble. To further assess the impact of uncertainties in the N flux model (*SI Appendix*, Fig. S5) on future nitrogen projections, we repeated the full analysis using a Monte Carlo approach where we sampled the uncertainty of the model illustrated in *SI Appendix*, Fig. S5*C* for each HUC8 watershed and for each climate model (Figs. 3 and 4) and each climate scenario (Fig. 4). We did so by randomly sampling the underlying conditional lognormal distribution and then aggregating the sampled nitrogen fluxes for all the HUC8 watersheds in a given region (*SI Appendix*, Fig. S8) and across the full CONUS (*SI Appendix*, Fig. S9). We repeated this process 1,000 times. Because deviations from the median behavior tend to largely cancel once HUC8 watersheds are aggregated to regional scales, however, the impact on the uncertainties presented in *SI Appendix*, Figs. S8 and S9 is minor, indicating the robustness of our future projections to model uncertainties.

### Calculation of *Q*_10_ and Its Uncertainty.

The fractional change in denitrification with a 10 °C rise in temperature ( $Q_{10}$ ) was calculated to compare the temperature sensitivity inferred here to those reported in bench-scale studies (*SI Appendix*, Table S1). For this calculation, we assumed that all nitrogen losses (i.e., nitrogen surplus minus riverine nitrogen loading) were due to denitrification. By keeping other covariates (i.e., annual precipitation, springtime extreme precipitation, nitrogen surplus, land cover fractions, and tile drainage fraction) at their median values of the training dataset, we calculated the nitrogen losses at different temperatures ranging from the minimum to the maximum annual temperature observed in the training dataset (i.e., −2 °C to 24 °C). Then, the $Q_{10}$ values were calculated as the ratio of nitrogen losses between (t+5) °C and (t-5) °C, with t ranging from 3 °C to 19 °C. We calculated the weighted average of $Q_{10}$ using the probability density function of observed temperature values in the training dataset and denoted the uncertainty of $Q_{10}$ as *Q*_10,19 °C_ to *Q*_10,3 °C_.

## Supplementary Material

Appendix 01 (PDF)

## Data Availability

Observed riverine nitrogen loading is available at the Water Quality Portal (WQP; https://www.waterqualitydata.us/) (67). Historical daily precipitation and temperature data are from the PRISM database (https://prism.oregonstate.edu/) (68). Nitrogen surplus data over the CONUS were collected from the TREND-nitrogen dataset (https://doi.pangaea.de/10.1594/PANGAEA.917583) (51). Land use data over the CONUS were calculated based on the NLCD2006 (https://www.mrlc.gov/data/nlcd-2006-land-cover-conus) (69). CMIP6 data were collected from the Google Cloud (https://console.cloud.google.com/marketplace/product/noaa-public/cmip6) (70). NHD data were collected from the United States Geological Survey (https://www.usgs.gov/national-hydrography) (71). Land Change Monitoring, Assessment, and Projection (LCMAP) data were collected from the United States Geological Survey (https://www.usgs.gov/special-topics/lcmap) (72). The WRTDS software (73) is available at https://rconnect.usgs.gov/EGRET/. The SDM downscaling code (74) is available at https://github.com/wegener-center/pyCAT. All other data are available in the main text and/or *SI Appendix*.
