# Supplementary material for: Warming may offset impact of precipitation changes on riverine nitrogen loading
Source: Proc Natl Acad Sci U S A. 2023 Aug 7;120(33):e2220616120. doi: 10.1073/pnas.2220616120 (PMC10438841; doi:10.1073/pnas.2220616120)
Supplement: Supplementary file 1 — Appendix 01 (PDF) [file pnas.2220616120.sapp.pdf]

PNAS

Supporting Information for
Warming may offset impact of precipitation changes on riverine nitrogen loading

Gang Zhao*, Julian Merder, Tristan C. Ballard, Anna M. Michalak

* Corresponding to Gang Zhao
Email: gzhao@carnegiescience.edu

This PDF file includes:

Figures S1 to S13
Tables S1 to S5
SI References

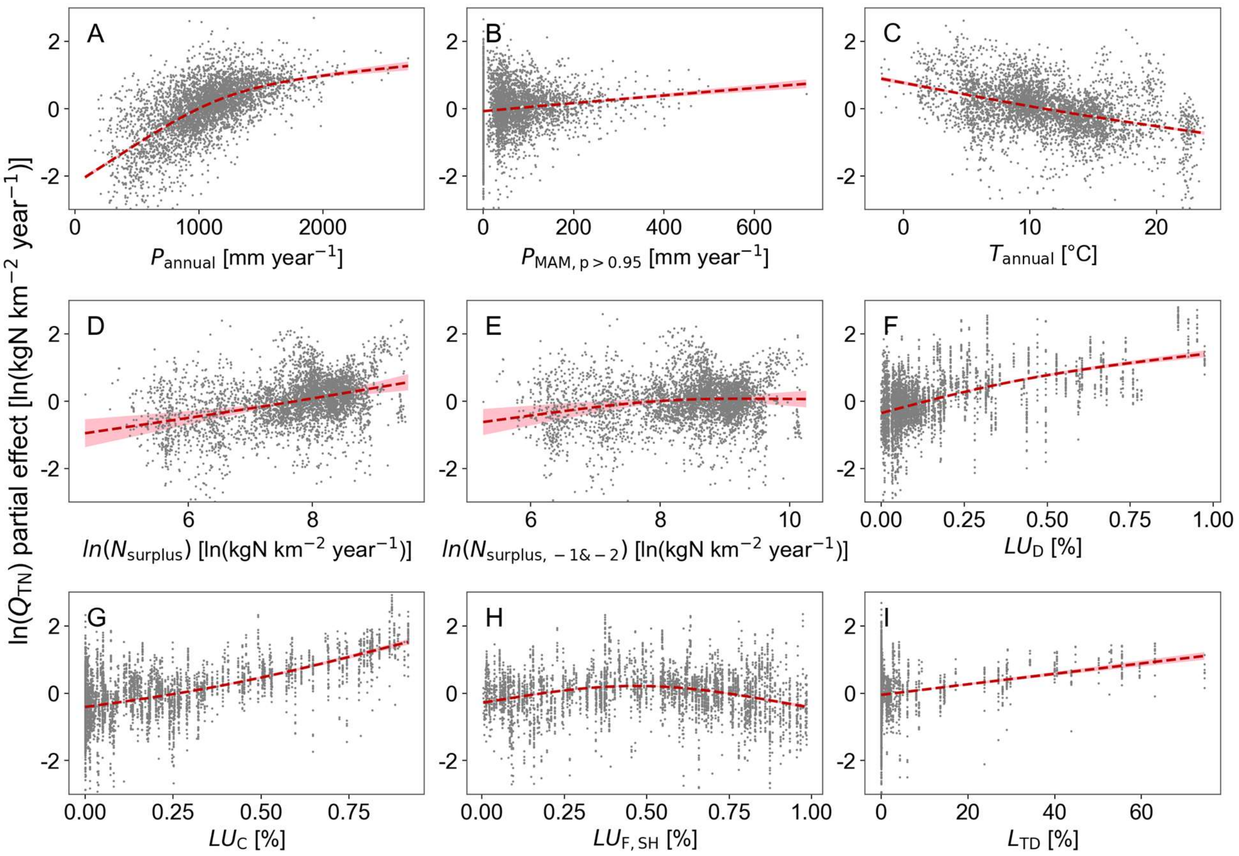

Fig. S1. Partial effect of selected covariates on the natural log of annual riverine nitrogen loading (ln(Q_{TN})) based on the fitted generalized additive model (GAM). The red line corresponds to the partial effect of the covariate and the shaded area indicates its estimated 95% confidence interval. The scatters represent the partial residuals. The zero value for the y-axis corresponds to the model intercept. The selected variables consist of (A) total annual precipitation (P_{annual}), (B) total spring-time (March, April, and May) precipitation that exceed 95th percentile of historical (1981-2010) values ($P_{MAM,p>0.95}$), (C) average annual temperature (T_{annual}), (D) natural-log-transformed N surplus [$ln(N_{surplus})$], (E) natural-log-transformed N surplus of previous 2 years [$ln(N_{surplus,-1\&-2})$], (F) percent coverage of developed land (LU_D), (G) percent coverage of cultivated land (LU_C), (H) percent coverage of forest and shrubland ($LU_{F,SH}$), and I percent of land with tile drainage (L_{TD}).

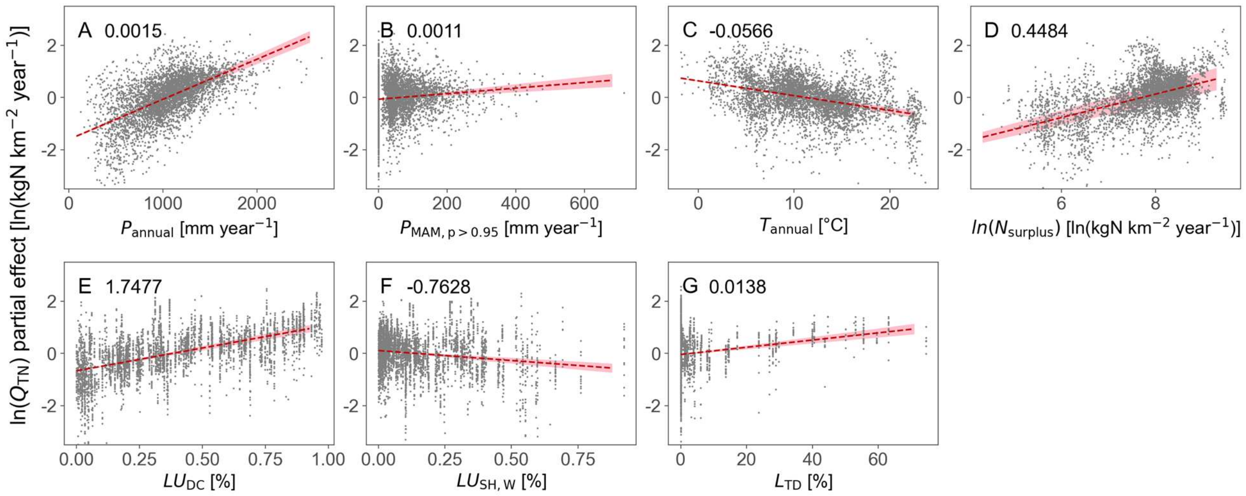

Fig. S2. Partial effect of selected covariates on the natural log of annual riverine nitrogen loading (ln(Q_{TN})) based on the fitted generalized linear model (GLM). The training of the GLM is based on the same dataset used in the generalized additive model (GAM). The numbers in the plot are the drift coefficients for each covariate. The red line corresponds to the partial effect of the covariate and the shaded area indicates its estimated 95% confidence interval. The scatters represent the partial residuals. The zero value for the y-axis corresponds to the model intercept. The selected variables consist of (A) total annual precipitation (P_{annual}), (B) total spring-time (March, April, and May) precipitation that exceed 95th percentile of historical (1981-2010) values ($P_{MAM,p>0.95}$), (C) average annual temperature (T_{annual}), (D) natural-log-transformed N surplus [$ln(N_{surplus})$], (E) percent coverage of developed land and cultivated land (LU_{DC}), (F) percent coverage of shrubland and wetland ($LU_{SH,W}$), and (G) percent of land with tile drainage (L_{TD}).

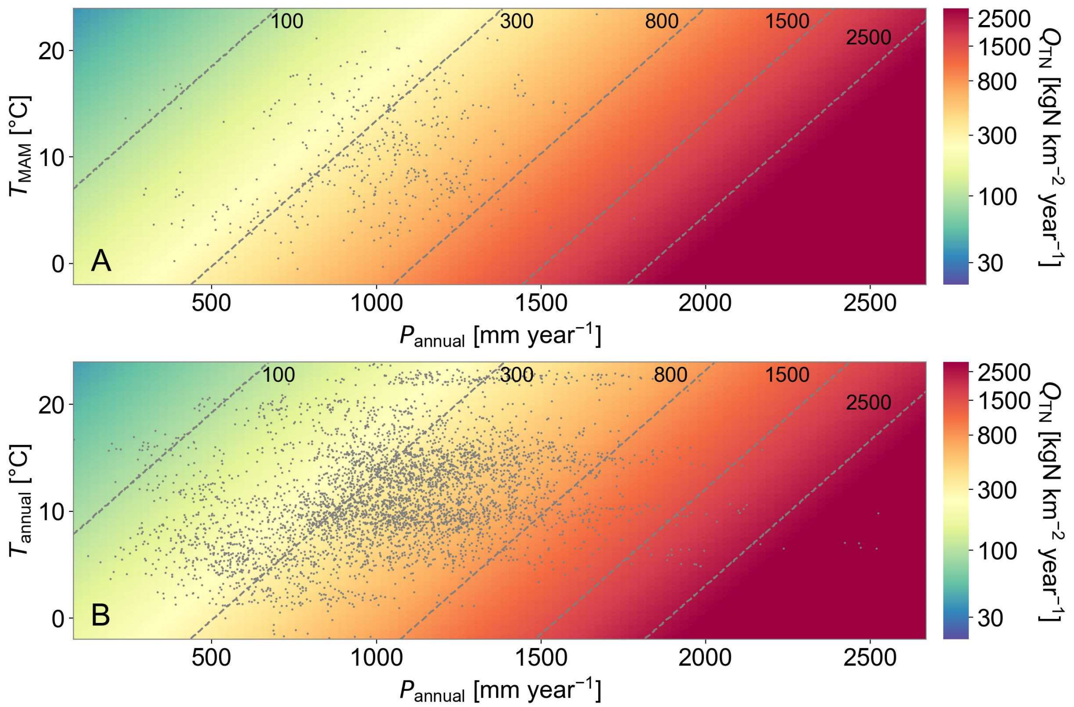

Fig. S3. Joint effects of precipitation and temperature on the natural log of annual riverine nitrogen loading (ln(Q_{TN})) for a linear model. (A) is based on the generalized linear model from Ballard et al. (GLM_B; Ballard et al. (1) and (B) is based on the generalized linear model (GLM) using the same training data as the generalized linear model (GAM). For the calculation of Q_{TN}, other covariates (e.g., nitrogen surplus and land cover) were held as the median of the training data. The scatter points represent the training data used for each model. P_{annual} represents total annual precipitation. T_{annual} represents average annual temperature and T_{MAM} represents average springtime (March, April, and May) temperature.

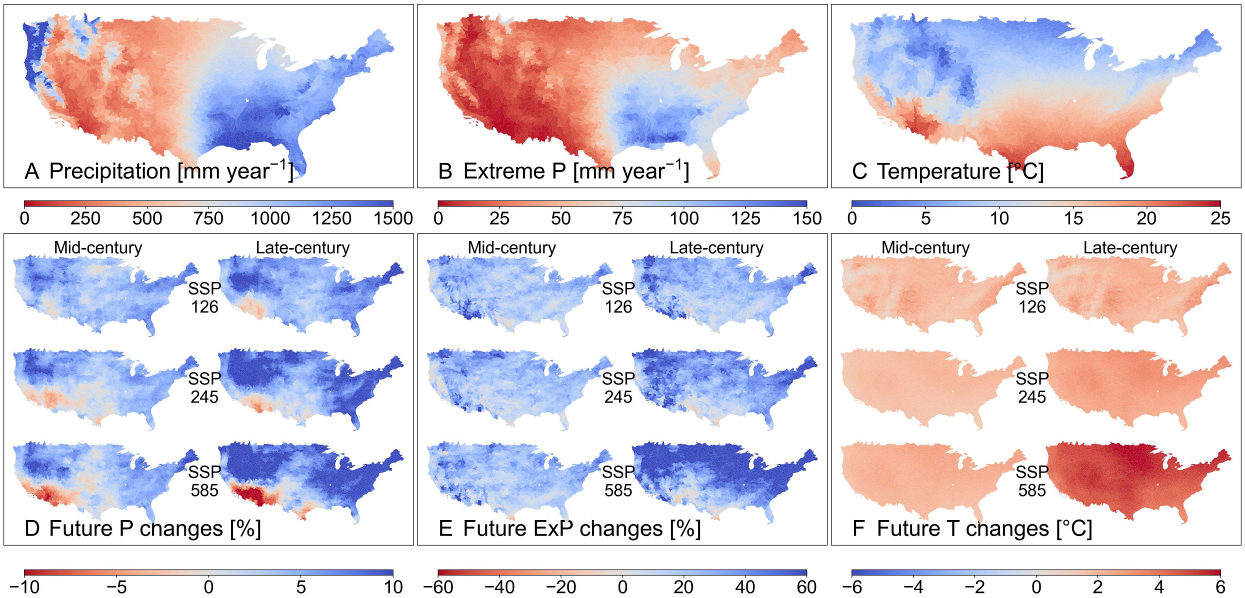

Fig. S4. Changes of annual precipitation, springtime extreme precipitation, and temperature in the future compared to the historical period. (A) Historical (1988-2017) average annual precipitation calculated as the ensemble median of the 16 climate models averaged over the SSP126 "sustainability," SSP245 "middle-of-the-road," and SSP585 "fossil-fueled-development" scenarios. (B) Historical (1988-2017) springtime extreme precipitation calculated as the ensemble median of the 16 climate models averaged over the three scenarios. (C) Historical (1988-2017) average annual temperature calculated as the ensemble median of the 16 climate models averaged over the three scenarios. (D) Projected future changes in annual precipitation for the mid- (2030-2059) and late-century (2070-2099) under three scenarios. (E) Projected future changes in springtime extreme precipitation during the two future periods under the three scenarios. (F) Projected future changes in annual temperature during the two future periods under the three scenarios. For the future change calculations, the historical period from 1988 to 2017 was used as the baseline and change values are shown as the ensemble median of the 16 climate models.

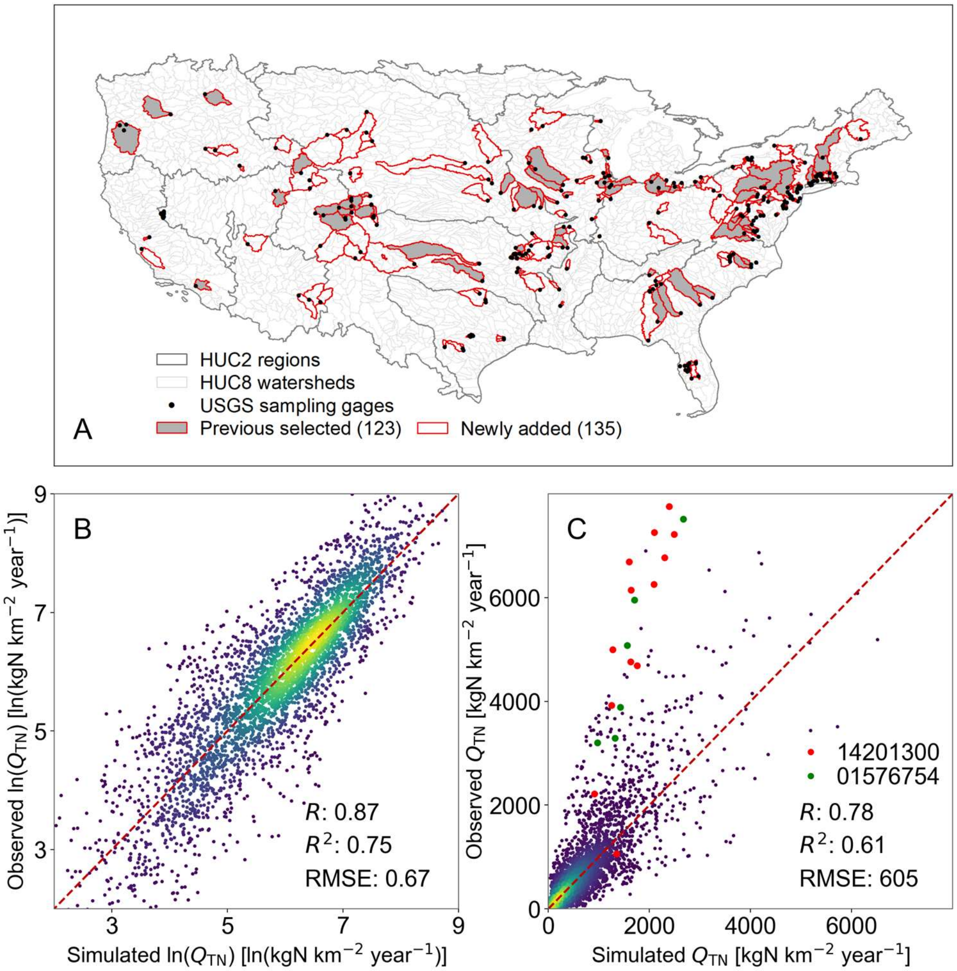

Fig. S5. Fitting of the generalized additive model for annual nitrogen loading. (A) The expansion of selected catchments that have observed annual nitrogen loading (Q_{TN}) relative to previous studies. (B) The comparison between simulated natural log of Q_{TN} and observed natural log of Q_{TN}. (C) The comparison between modeled Q_{TN} and observed Q_{TN}. The selected 258 watersheds consist of the 123 watersheds that were used in Ballard et al. (1) and the 135 newly selected watersheds in this study based on the expanded period of record. The observed Q_{TN} was calculated using the weighted regressions on time, discharge, and season (WRTDS) tool and the simulated Q_{TN} was calculated by the fitted generalized additive model (GAM). The clustering of underestimation for several points of Q_{TN} mainly originate from two small, nested watersheds (USGS Gage ID 01576754 with the contributing area of 20 km^2 and 14201300 with a contributing area of 1200 km^2). However, the biases in two specific watersheds are not representative and therefore unlikely to affect the overall performance of the model.

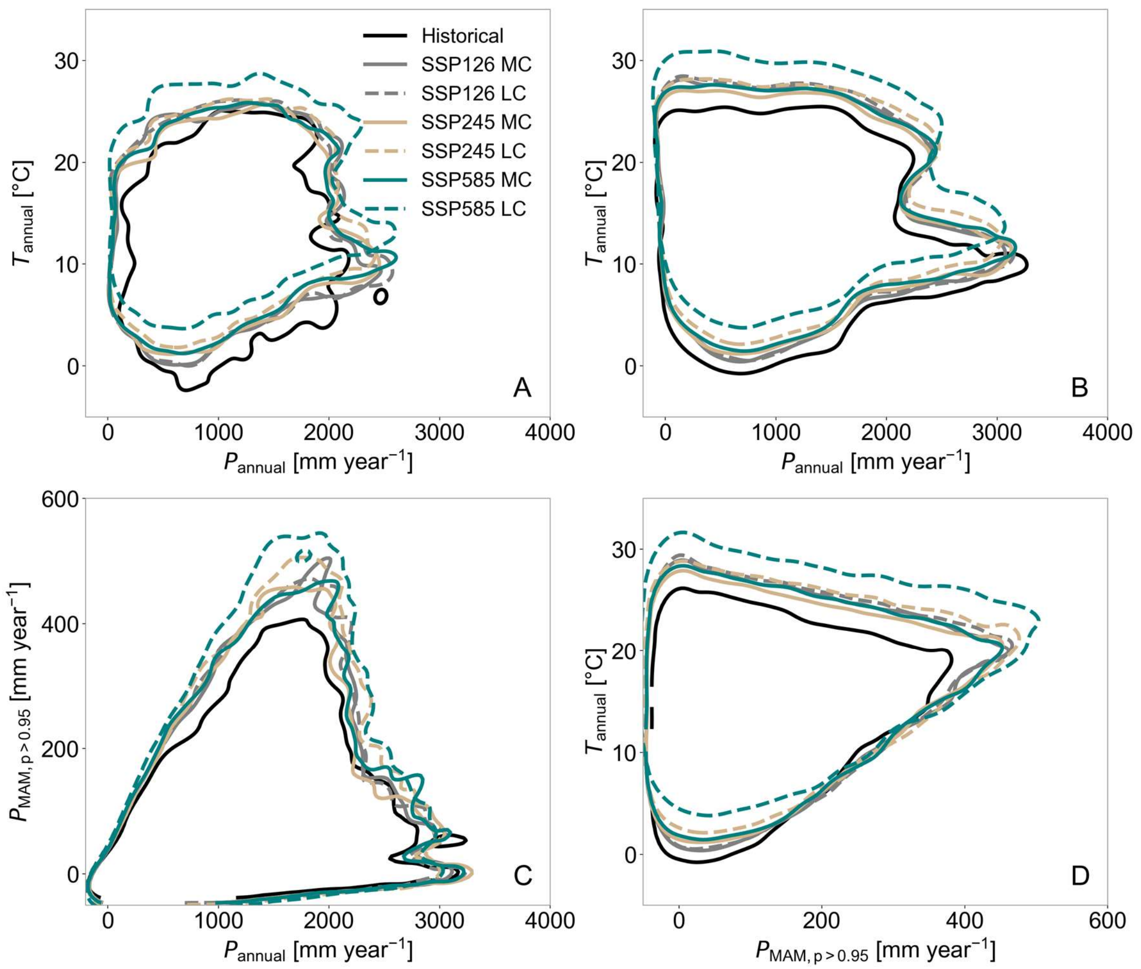

Fig. S6. Future changes of precipitation and temperature relative to the historical period. (A) Two dimensional distribution (0.01 iso-proportion of the density) of annual precipitation and annual temperature for the catchments that were used as part of the training dataset. (B) Two dimensional distribution of annual precipitation and annual temperature for all the 8-digit hydrologic unit (HUC8) watersheds. (C) Two dimensional distribution of annual precipitation and springtime extreme precipitation for all the 8-digit hydrologic unit (HUC8) watersheds. (D) Two dimensional distribution of springtime extreme precipitation and annual temperature for all the 8-digit hydrologic unit (HUC8) watersheds. The locations of the training catchments and all HUC8 watersheds can be found in Fig. S5. MC represents the "Mid-century" and LC represents "Late-century". The historical values were collected from the Precipitation-elevation Regressions on Independent Slopes Model (PRISM) dataset and the future values were calculated using the bias-corrected climate model outputs (see Methods). P_{annual} represents the total annual precipitation. $P_{MAM,p>0.95}$ represents the total springtime (March, April, and May) precipitation that exceed 95th percentile of historical (1981-2010) values ($P_{MAM,p>0.95}$). T_{annual} represents the average annual temperature.

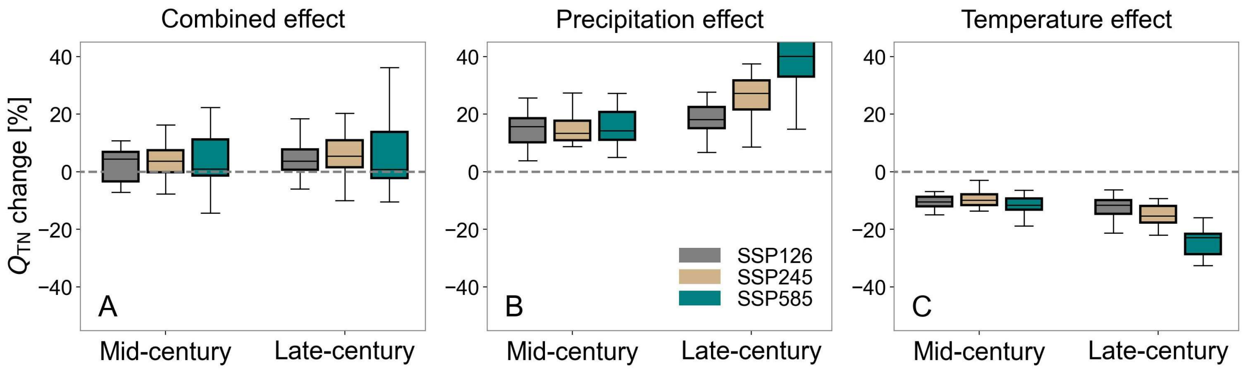

Fig. S7. Changes in annual nitrogen loading for the CONUS in response to future precipitation and temperature changes based on the generalized linear model from Ballard et al. (1) (GLM_B). (A) Changes in Q_{TN} in response to the changes in total annual precipitation, springtime extreme precipitation, and annual temperature. (B) Changes in annual nitrogen loading (Q_{TN}) in response only to the changes in total annual precipitation and springtime extreme precipitation. (C) Changes in Q_{TN} in response only to the changes in annual temperature. The changes in Q_{TN} were calculated as the relative change between the mid- (2020-2049) and late-century (2070-2099) and the historical (1988-2017) periods for each climate model. The spread in the boxplots represents variability in projected change across the 16-model ensemble.

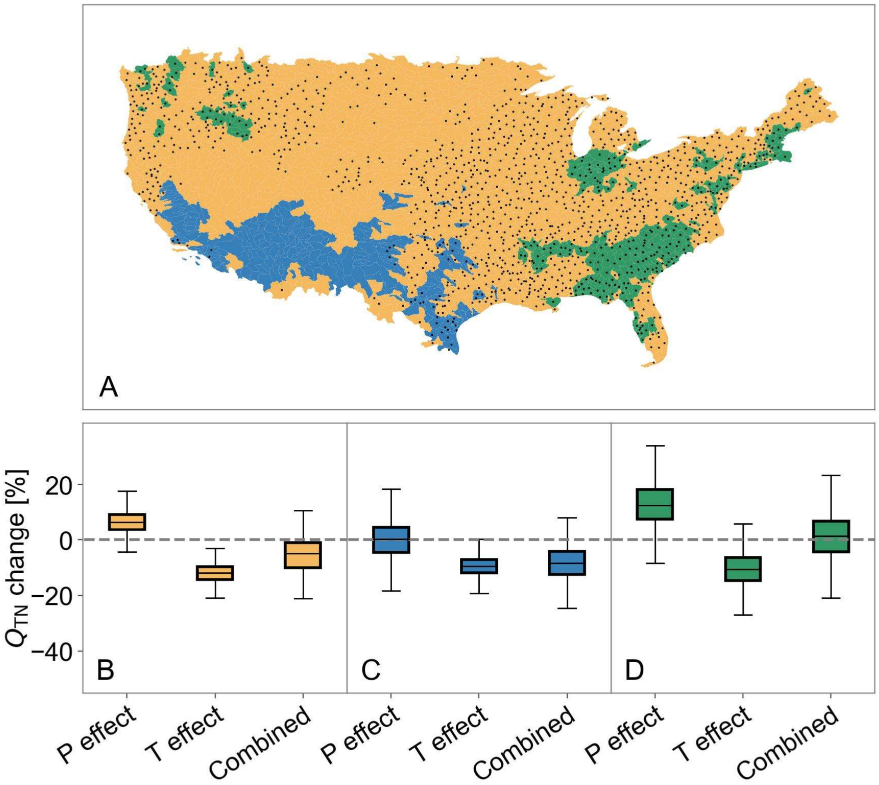

Fig. S8. Tradeoff or compounding effects of precipitation and temperature changes on annual nitrogen loading by considering uncertainties from both climate models and GAM. This figure is the same as Fig. 3 in the main text but incorporates modeling uncertainty from GAM.

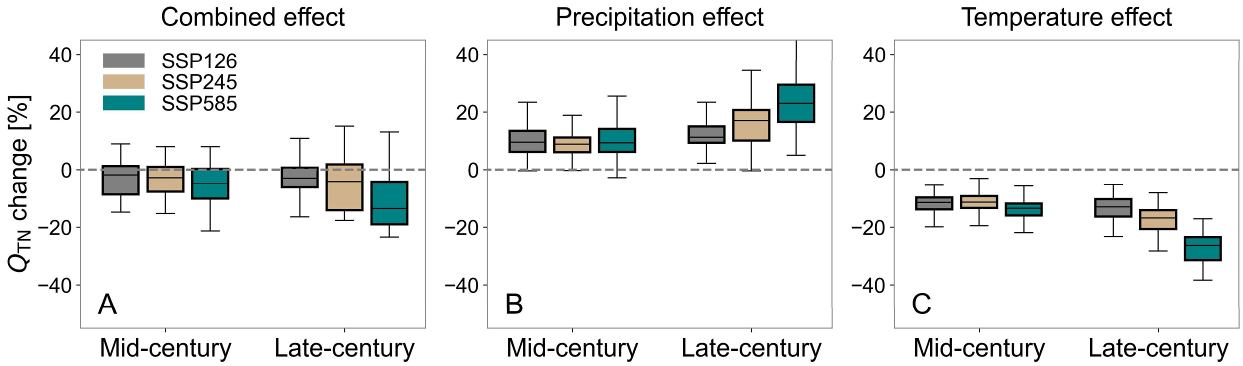

Fig. S9. Changes in annual nitrogen loading for the CONUS in response to future precipitation and temperature changes by considering uncertainties from both climate models and GAM. This figure is the same as Fig. 4 in the main text but incorporates modeling uncertainty from GAM.

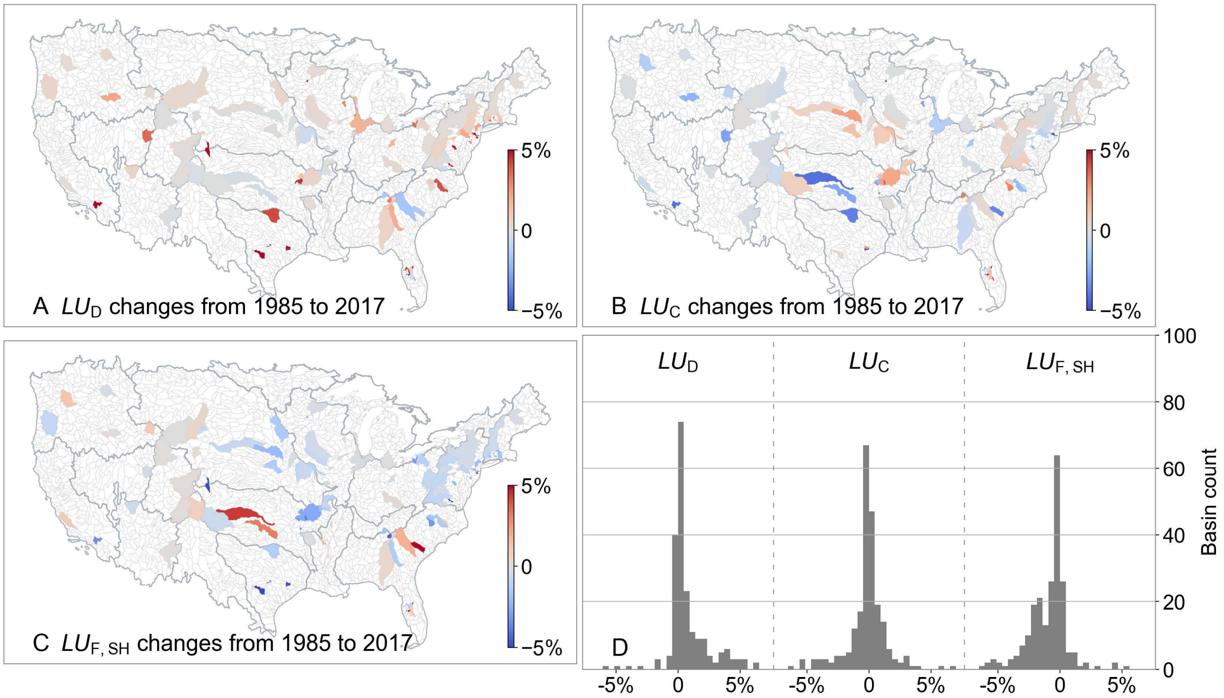

Fig. S10. Land cover change from 1985 to 2017 in the 258 training catchments. Geographic patterns are presented in (A) for developed area (LU_D), (B) for cropland (LU_C), and (C) for forest and shrubland ($LU_{F,SH}$), while panel (D) presents histograms of changes in LU_D, LU_C, and $LU_{F,SH}$ for the 258 catchments. The percentage change was calculated as the land cover percentage in 2017 minus the land cover percentage in 1985. Dynamic land cover data were collected from the Land Change Monitoring, Assessment, and Projection (LCMAP) (2).

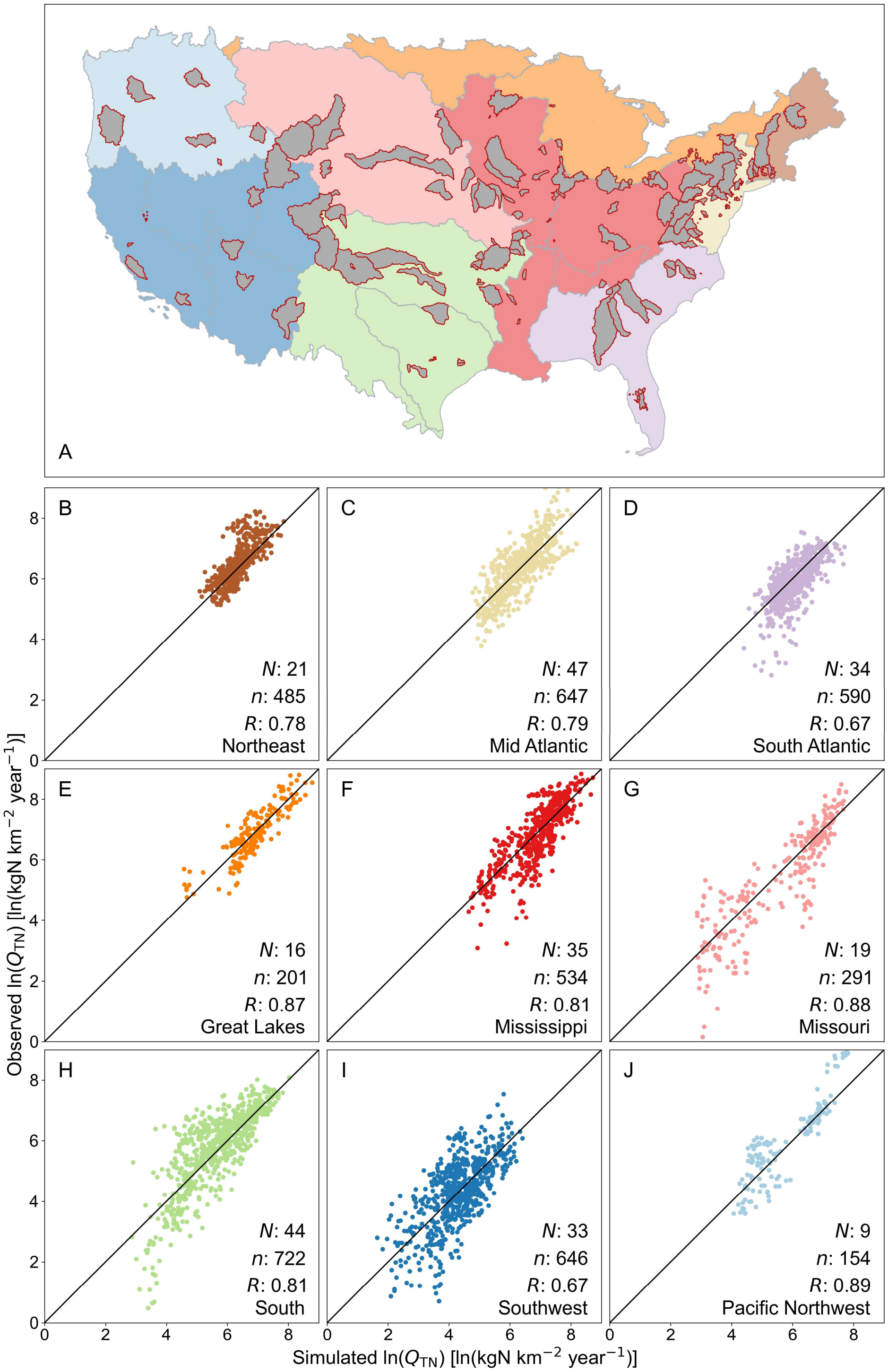

Fig. S11. Equivalent to Figure S5B but binning the observed and simulated nitrogen loads by region to show that the model reproduces space-time variability within regions and not simply between regions. (A) The nine regions based on 2-digit hydrologic units (HUC2). (B)-(J) The comparison between simulated natural log of Q_{TN} and observed natural log of Q_{TN} for each region. N represents the number of catchments in each region and totals 258 for the CONUS while n represents the number of annual TN loading observations across all the catchments in each region and totals 4270 for the CONUS.

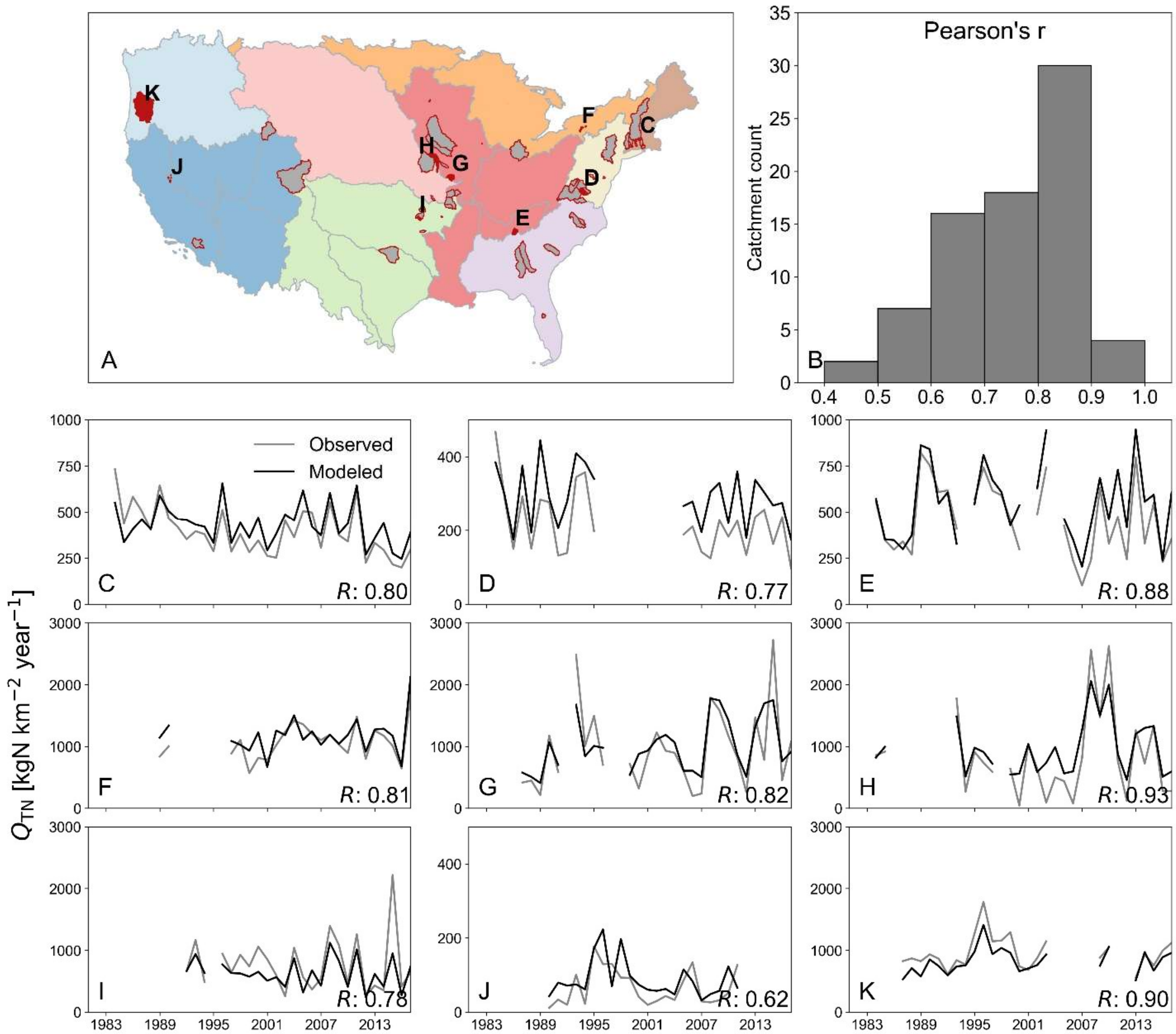

Fig. S12. Examples of how the model reproduces observed temporal variability for individual catchments. Among the 258 catchments in our training dataset, there are 78 with at least 20 years of observed nitrogen loading data. We show the locations of these catchments in panel (A). Panel (B) shows the histogram of the Pearson's r value between simulated and observed annual nitrogen loading (Q_{TN}) for these 78 catchments. This demonstrates that the model can capture temporal variability for individual catchments. Panels (C)-(K) show time series from one catchment for each of the large regions in (A). The catchment corresponding to the median area among the eligible catchments within each area was chosen for illustration and also marked in red and labeled them in panel (A).

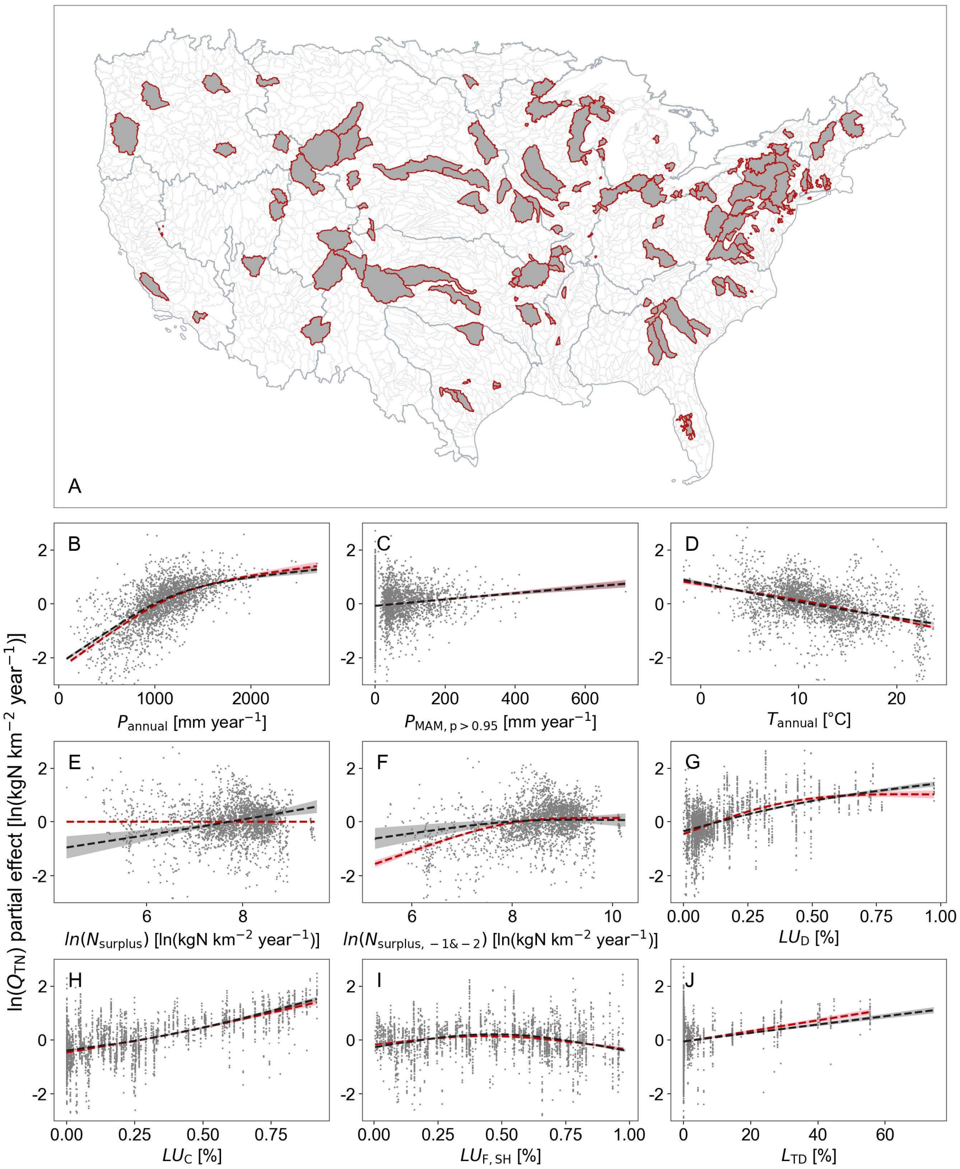

Fig. S13. Partial effect of selected covariates on the natural log of annual riverine nitrogen loading ($ln(Q_{TN})$) after removing nested catchments. (A) The remaining 196 basins after removing fully nested catchments. (B)-(J) Partial effects of selected covariates on the natural log of annual riverine nitrogen loading based on generalized additive model (GAM). The red lines show the partial effect from the retrained model and the black lines show the partial effect from the original model (i.e., Fig. S1).

Table S1. Q10 values and optimal temperature of the denitrification process from incubation experiments. Studies that reported multiple values are listed in multiple rows. The incubation temperature represents the temperature range in degrees centigrade for which the experiment was implemented. The sampling temperature represents the temperature of the sample (if recorded during sampling) or air temperature from the ERA5 dataset (if not recorded during sampling). Optimal temperature represents the temperature at which the highest rate of denitrification was observed. The slash sign indicates data not measured.

Longitude	Latitude	Environment	Process	Q10	Incubation T	Sampling T	Optimal T	Reference
-84.77	29.70	Near-shore sediment	Denitrification	3.8	0-40	29.9	36	(3)
-84.87	29.66	Near-shore sediment	Denitrification	5	0-40	31.8	35	(3)
8.44	55.02	Near-shore sediment	Denitrification	2	0-40	19	34	(3)
8.44	55.02	Near-shore sediment	Denitrification	2.5	0-40	5.3	26	(3)
14.03	78.27	Near-shore sediment	Denitrification	2.3	0-40	6.8	21	(3)
118.10	24.63	Near-shore sediment	Denitrification	1.90	0-35	36.5	35	(4)
118.10	24.28	Near-shore sediment	Denitrification	2.20	0-35	34.6	35	(4)
118.10	24.42	Near-shore sediment	Denitrification	2.70	0-35	32.9	35	(4)
118.10	24.47	Near-shore sediment	Denitrification	2.70	0-35	31.6	35	(4)
118.10	24.45	Near-shore sediment	Denitrification	2.20	0-35	30.7	35	(4)
118.10	24.61	Near-shore sediment	Denitrification	1.90	0-35	30	30	(4)
118.10	24.44	Near-shore sediment	Denitrification	3.10	0-35	24	30	(4)
118.10	24.46	Near-shore sediment	Denitrification	3.20	0-35	22.3	30	(4)
118.10	24.61	Near-shore sediment	Denitrification	2.70	0-35	17	25	(4)
118.10	24.28	Near-shore sediment	Denitrification	2.20	0-35	16.5	35	(4)
118.10	24.59	Near-shore sediment	Denitrification	3.50	0-35	16.2	30	(4)
118.10	24.19	Near-shore sediment	Denitrification	2.60	0-35	16.3	35	(4)
/	/	Water-logged Soil	Denitrification	2	15-35	/	/	(5)
28.76	73.83	Marine sediment	Denitrification	2.4	-5-40	-0.22	24	(6)
-71.34	41.53	Temperate marine sediment	Denitrification	/	0-60	12.25	18-35	(7)
-2.57	55.88	Grassland soil	N_2O production	8.9	12-18	6.9	>18	(8)
-2.57	55.88	Grassland soil	N_2O production	50	5-12	6.9	>18	(8)
-2.57	55.88	Arable soil	N_2O production	2.3	12-18	6.9	>18	(8)
-2.57	55.88	Arable soil	N_2O production	3.7	5-12	6.9	>18	(8)
35.81	59.97	Agricultural Soil	N_2O production	4.186	4-37	2.3	25-37	(9)
5.64	51.97	Vegetated ditch	Denitrification	4.411	10-25	9.4	>25	(10)
1.77	43.04	Phototrophic river biofilms	Denitrification	7	1.1-30.9	/	/	(11)

Longitude	Latitude	Environment	Process	Q10	Incubation T	Sampling T	Optimal T	Reference
9.00	56.00	Groundwater sediment	Denitrification	1.8	9.1-28.6	9	30-40	(12)
9.95	51.53	Agricultural Soil	N_2O production	3.7	13.5-23.5	/	/	(13)
-6.54	52.86	Grazed pastures	Denitrification	5.8	10-25	/	/	(14)
-121.73	38.53	Agricultural Soil	N_2O production	1.2	5-15	/	/	(15)
-121.73	38.53	Agricultural Soil	N_2O production	2.80	5-15	/	/	(15)
-121.73	38.53	Agricultural Soil	N_2O production	5.4	5-15	/	/	(15)
-121.73	38.53	Agricultural Soil	N_2O production	2.20	10-20	/	/	(15)
-121.73	38.53	Agricultural Soil	N_2O production	4.20	10-20	/	/	(15)
-121.73	38.53	Agricultural Soil	N_2O production	20†	10-20	/	/	(15)
-121.73	38.53	Agricultural Soil	N_2O production	1.40	15-25	/	/	(15)
-121.73	38.53	Agricultural Soil	N_2O production	3.40	15-25	/	/	(15)
-121.73	38.53	Agricultural Soil	N_2O production	33†	15-25	/	/	(15)
-2.85	56.04	Arable soil	Autotrophic N_2O production	2.1	2-25	/	/	(16)
-2.85	56.04	Arable soil	Autotrophic N_2O production	2	25-40	/	/	(16)
-2.85	56.04	Arable soil	Heterotrophic N_2O production	2	2-25	/	/	(16)
-2.85	56.04	Arable soil	Heterotrophic N_2O production	3.4	25-40	/	/	(16)
-2.85	56.04	Arable soil	N_2 production	8.9	2-25	/	/	(16)
-2.85	56.04	Arable soil	N_2 production	9.7	25-40	/	/	(16)
-2.85	56.04	Forest soil	N_2 production	2.5	2-25	/	/	(16)
-2.85	56.04	Forest soil	N_2 production	2.7	25-40	/	/	(16)
19.11	64.96	Lake sediment	Denitrification	1.69	4-25	0.08	>25	(17)
19.11	64.96	Lake sediment	N_2O production	1.77	4-25	0.08	>25	(17)
5.65	51.99	Wetland sediment	Denitrification	2.6	11-25	/	/	(18)
25.37	64.84	River sediment	N_2O production	3.1	5-20	2.3	>20	(19)
140.19	36.23	River sediment	Denitrification	/	5-35	16.1	>35	(20)

†Water content was 90%, which may create more anaerobic conditions.

Table S2. Comparison of covariates included in model implemented in Sinha and Michalak (21), Ballard et al. (1), and this study. NANI represents net anthropogenic nitrogen inputs (see Methods) and the definitions of the variables can be found in Supplementary Table S3.

Study	Number of basins	Temporal coverage	Excess N source	Number of observations	Covariates selected
Sinha and Michalak (21)†	70	1987, 1992, 1997, 2002, 2007	NANI (22)	242	f_{NANI}, P_{annual}, $P_{MAM,p>0.95}$, LU_W, $LU_{F,SH}$
Ballard et al. (1)‡	123	1987, 1992, 1997, 2002, 2007, 2012	NANI (23)	440	f_{NANI}, P_{annual}, $P_{MAM,p>0.95}$, T_{MAM}, LU_W, $LU_{F,SH}$
This study§	258	Every year from 1981 to 2017	N-surplus (24)	4270	$ln(N_{surplus})$, $ln(N_{surplus,-1\&-2})$, P_{annual}, $P_{MAM,p>0.95}$, T_{annual}, LU_D, LU_C, $LU_{F,SH}$, L_{TD}

† A generalized linear model with the formulation of $ln(Q_{TN}) = 3.01 + 0.3742f_{NANI} + 0.0014P_{annual} + 0.0033P_{MAM,p>0.95} - 0.0529LU_w - 0.0220LU_{F,SH}$, where the definition of covariates can be found in Table S3.

‡ A generalized linear model with the formulation of $ln(Q_{TN}) = 2.58 + 0.466f_{NANI} + 0.00160P_{annual} + 0.00247P_{MAM,p>0.95} - 0.0585T_{MAM} - 0.0438LU_w - 0.0229LU_{F,SH}$, where the definition of covariates can be found in Table S3.

§ A generalized additive model, whose partial effects are illustrated in Fig. S1.

Table S3. Summary of all potential model covariates (Category, Variable(s) considered, Variable description(s), Number of variables, Considered), the constraints used in the model selection procedure in terms of the maximum number of variables allowed from each category (Number of variables, Allowed), and the variables included in the final model (Selected variables).

Category	Variable(s) considered	Variable description(s)	Number of variables		Data source	Selected variables
			Considered	Allowed		
Nitrogen surplus (current year)	$N_{Surplus}$; $ln(N_{Surplus})$	Nitrogen surplus [kg-N km-2 yr-1] and log-scale nitrogen surplus for current year.	2	1	*(24)*	$ln(N_{Surplus})$
Nitrogen surplus (previous years)	$N_{Surplus,-1}$; $ln(N_{Surplus,-1})$; $N_{Surplus,-2}$; $ln(N_{Surplus,-2})$; $N_{Surplus,-1\&-2}$; $ln(N_{Surplus,-1\&-2})$;	Nitrogen surplus and log-scale nitrogen surplus for prior year (-1), two year prior (-2), and cumulative over two prior years (-1&-2).	6	2†	*(24)*	$ln(N_{Surplus,-1\&-2})$
Precipitation	P_{annual}; P_{MAM}	Total annual (Annual) and March, April, and May (MAM) precipitation [mm]	2	2	*PRISM (25)*	P_{annual}
Extreme precipitation	$P_{p>0.90}$; $P_{p>0.95}$; $P_{p>0.99}$	Total precipitation on days with precipitation above the 90th, 95th or 99th percentile‡ (calculated based on 30 years of daily precipitation amounts from 1981-2010) [mm]	3	1	*PRISM (25)*	$P_{MAM,p>0.95}$
	$P_{MAM,p>0.90}$; $P_{MAM,p>0.95}$; $P_{MAM,p>0.99}$	Total precipitation (mm) in March, April & May on days with precipitation greater than 90th, 95th or 99th percentile (calculated based on 30 years of daily precipitation from 1981-2010)	3			

	$P_{MAM,p(MAM)>0.90}$; $P_{MAM,p(MAM)>0.95}$; $P_{MAM,p(MAM)>0.99}$	Total precipitation (mm) in March, April & May on days with precipitation greater than 90th, 95th or 99th percentile (calculated based on 30 yrs of daily precipitation in March, April & May from 1981-2010)	3			
Temperature	T_{annual}; T_{MAM}	Average annual (Annual) and March, April, and May (MAM) temperature [°C]	2	1	*PRISM (25)*	T_{annual}
Land use	LU_D; LU_C; LU_F; LU_{SH}; LU_W; $LU_{D,C}$; $LU_{D,F}$; $LU_{D,SH}$; $LU_{D,W}$; $LU_{C,F}$; $LU_{C,SH}$; $LU_{C,W}$; $LU_{F,SH}$; $LU_{F,W}$; $LU_{SH,W}$; $LU_{D,C,F}$; $LU_{D,C,SH}$; $LU_{D,C,W}$; $LU_{D,F,SH}$; $LU_{D,F,W}$; $LU_{D,SH,W}$; $LU_{C,F,SH}$; $LU_{C,F,W}$; $LU_{C,SH,W}$; $LU_{F,SH,W}$; $LU_{D,C,F,SH}$; $LU_{D,C,F,W}$; $LU_{D,F,SH,W}$; $LU_{D,C,SH,W}$; $LU_{C,F,SH,W}$	Percentage of land use classified as Developed (D), Cultivated (C), Forest (F), Shrubland & Herbaceous (SH) and Wetlands (W) and various combinations of two, three and four land use categories [%]	30	4§	*NLCD2006 (26)*	LU_D; LU_C; $LU_{F,SH}$
Tile drainage	L_{TD}	Percentage of land with tile drainage	1	1	*(27)*	L_{TD}
**TOTAL**			**52**	**12**		**9**

† Nitrogen input variables from previous years are only allowed if at least one variable from the nitrogen input in the current year is included.

‡ 90th, 95th, and 99th percentiles were defined during wet days in the 1981-2010 time-period. In this study wet days were defined as days with precipitation greater than 1.0 mm.

§ Any single land use category can only be represented once in the model either as an individual or binned category, and a maximum of four land use categories can be represented either as single or binned categories.

Table S4. The 16 climate models that were used in the quantification of future annual nitrogen loading. Modeled daily precipitation and monthly temperature were collected from the Coupled Model Inter-comparison Project phase 6 (CMIP6). Model selection was based on data availability on the Google Cloud Storage (GCP; console.cloud.google.com/storage/browser/cmip6) at the time of this work. Models with an earth system model (ESM) component were prioritized if a modeling group had multiple options (e.g., GFDL-CM4 vs. GFDL-ESM4)

Model name	Modeling center	Nominal resolution
BCC-CSM2-MR	Beijing Climate Center, China Meteorological Administration, China	1.125°x1.125°
CanESM5	Canadian Centre for Climate Modelling and Analysis, Canada	2.813°x2.789°
IITM-ESM	Centre for Climate Change Research-Indian Institute of Tropical Meteorology, India	1.875°x1.904°
CNRM-ESM2-1	Center National de Recherches Météorologiques–Center Européen de Recherche et de Formation Avancée en Calcul Scientifique, France	1.406°x1.400°
ACCESS-ESM1-5	Commonwealth Scientific and Industrial Research Organisation, Australia	1.875°x1.250°
MPI-ESM1-2-HR	German Climate Computing Centre, Germany	0.938°x0.935°
EC-Earth3-Veg	EC-Earth-Consortium, Europe	0.703°x0.702°
INM-CM5-0	Institute for Numerical Mathematics, Russia	2.000°x1.500°
IPSL-CM6A-LR	L'Institut Pierre-Simon Laplace, France	2.500°x1.268°
MIROC-ES2L	Model for Interdisciplinary Research on Climate, Japan	2.813°x2.789°
HadGEM3-GC31-LL	Met Office Hadley Center, United Kingdom	1.875°x1.250°
UKESM1-0-LL	Met Office Hadley Center, United Kingdom	1.875°x1.250°
MRI-ESM2-0	Meteorological Research Institute, Japan	1.125°x1.121°
KACE-1-0-G	National Institute of Meteorological Sciences-Korea Meteorological Administration, South Korea	1.875°x1.250°
GFDL-ESM4	NOAA/Geophysical Fluid Dynamics Laboratory, USA	1.250°x1.000°
NESM3	Nanjing University of Information Science and Technology, China	1.875°x1.865°

Table S5. Comparison of watershed areas, total lengths of all streams within each watershed, and average in-stream travel times between the 258 training catchments and the 2087 HUC8 watersheds. Stream length was calculated using the NHD dataset (28) and in-stream travel time was calculated based on Allen et al. (29).

		25th quantile	Median	75th quantile
**Watershed area [km^2]**	**Training dataset**	250	1360	7980
	**HUC8**	2300	3360	4910
**Stream length [km]**	**Training dataset**	410	1980	15,620
	**HUC8**	3400	5800	10,360
**Travel time [hours]**	**Training dataset**	8.9	25.2	58.0
	**HUC8**	21.2	32.9	57.7

SI References

1. T. C. Ballard, E. Sinha, A. M. Michalak, Long-term changes in precipitation and temperature have already impacted nitrogen loading. *Environmental Science & Technology* **53**, 5080-5090 (2019).
2. J. F. Brown *et al.*, Lessons learned implementing an operational continuous United States national land change monitoring capability: The Land Change Monitoring, Assessment, and Projection (LCMAP) approach. *Remote Sensing of Environment* **238**, 111356 (2020).
3. A. Canion *et al.*, Temperature response of denitrification and anammox reveals the adaptation of microbial communities to in situ temperatures in permeable marine sediments that span 50° in latitude. *Biogeosciences* **11**, 309-320 (2014).
4. E. Tan *et al.*, Warming stimulates sediment denitrification at the expense of anaerobic ammonium oxidation. *Nature Climate Change* **10**, 349-355 (2020).
5. G. Stanford, S. Dzienia, R. A. Vander Pol, Effect of Temperature on Denitrification Rate in Soils. *Soil Science Society of America Journal* **39**, 867-870 (1975).
6. S. Rysgaard, R. N. Glud, N. Risgaard-Petersen, T. Dalsgaard, Denitrification and anammox activity in Arctic marine sediments. *Limnology and Oceanography* **49**, 1493-1502 (2004).
7. L. D. Brin, A. E. Giblin, J. J. Rich, Similar temperature responses suggest future climate warming will not alter partitioning between denitrification and anammox in temperate marine sediments. *Global Change Biology* **23**, 331-340 (2017).
8. K. E. Dobbie, K. A. Smith, The effects of temperature, water-filled pore space and land use on N2O emissions from an imperfectly drained gleysol. *European Journal of Soil Science* **52**, 667-673 (2001).
9. G. Braker, J. Schwarz, R. Conrad, Influence of temperature on the composition and activity of denitrifying soil communities. *FEMS Microbiology Ecology* **73**, 134-148 (2010).
10. A. J. Veraart, J. J. M. de Klein, M. Scheffer, Warming Can Boost Denitrification Disproportionately Due to Altered Oxygen Dynamics. *Plos One* **6**, e18508 (2011).
11. S. Boulêtreau, E. Salvo, E. Lyautey, S. Mastrorillo, F. Garabetian, Temperature dependence of denitrification in phototrophic river biofilms. *Science of The Total Environment* **416**, 323-328 (2012).
12. C. Juncher Jørgensen, O. S. Jacobsen, B. Elberling, J. Aamand, Microbial Oxidation of Pyrite Coupled to Nitrate Reduction in Anoxic Groundwater Sediment. *Environmental Science & Technology* **43**, 4851-4857 (2009).
13. A. Sänger, D. Geisseler, B. Ludwig, Effects of moisture and temperature on greenhouse gas emissions and C and N leaching losses in soil treated with biogas slurry. *Biology and Fertility of Soils* **47**, 249-259 (2011).
14. M. Abdalla, M. Jones, P. Smith, M. Williams, Nitrous oxide fluxes and denitrification sensitivity to temperature in Irish pasture soils. *Soil Use and Management* **25**, 376-388 (2009).
15. I. N. Kurganova, V. O. Lopes de Gerenyu, Effect of the temperature and moisture on the N2O emission from some arable soils. *Eurasian Soil Science* **43**, 919-928 (2010).
16. S. Castaldi, Responses of nitrous oxide, dinitrogen and carbon dioxide production and oxygen consumption to temperature in forest and agricultural light-textured soils determined by model experiment. *Biology and Fertility of Soils* **32**, 67-72 (2000).
17. M. Myrstener, A. Jonsson, A.-K. Bergström, The effects of temperature and resource availability on denitrification and relative N2O production in boreal lake sediments. *Journal of Environmental Sciences* **47**, 82-90 (2016).
18. J. J. M. de Klein, C. C. Overbeek, C. Juncher Jørgensen, A. J. Veraart, Effect of Temperature on Oxygen Profiles and Denitrification Rates in Freshwater Sediments. *Wetlands* **37**, 975-983 (2017).
19. H. Silvennoinen, A. Liikanen, J. Torssonen, C. F. Stange, P. J. Martikainen, Denitrification and N_2o Effluxes in the Bothnian Bay (Northern Baltic Sea) River Sediments as Affected by Temperature under Different Oxygen Concentrations. *Biogeochemistry* **88**, 63-72 (2008).
20. S. Zhou, S. Borjigin, S. Riya, A. Terada, M. Hosomi, The relationship between anammox and denitrification in the sediment of an inland river. *Science of The Total Environment* **490**, 1029-1036 (2014).

21. E. Sinha, A. M. Michalak, Precipitation dominates interannual variability of riverine nitrogen loading across the continental United States. *Environmental Science & Technology* **50**, 12874-12884 (2016).
22. B. Hong, D. P. Swaney, R. W. Howarth, A toolbox for calculating net anthropogenic nitrogen inputs (NANI). *Environmental Modelling & Software* **26**, 623-633 (2011).
23. B. Hong, D. P. Swaney, R. W. Howarth, Estimating net anthropogenic nitrogen inputs to US watersheds: comparison of methodologies. *Environmental Science & Technology* **47**, 5199-5207 (2013).
24. D. Byrnes, K. Van Meter, N. Basu, Long-Term Shifts in US Nitrogen Sources and Sinks Revealed by the New TREND-Nitrogen Data Set (1930–2017). *Global Biogeochemical Cycles* **34**, e2020GB006626 (2020).
25. C. Daly *et al.*, Physiographically sensitive mapping of climatological temperature and precipitation across the conterminous United States. *International Journal of Climatology: a Journal of the Royal Meteorological Society* **28**, 2031-2064 (2008).
26. C. Homer *et al.*, Conterminous United States land cover change patterns 2001–2016 from the 2016 national land cover database. *ISPRS Journal of Photogrammetry and Remote Sensing* **162**, 184-199 (2020).
27. Z. Sugg, Assessing US Farm Drainage: Can GIS lead to better estimates of subsurface drainage extent? *World Resources Institute, Washington, DC, 20002* (2007).
28. U.S. Geological Survey, National Hydrography Dataset (ver. USGS National Hydrography Dataset Best Resolution (NHD) (published 20191002)), accessed Mar 1, 2023 at URL https://www.usgs.gov/national-hydrography/access-national-hydrography-products. (2019).
29. G. H. Allen, C. H. David, K. M. Andreadis, F. Hossain, J. S. Famiglietti, Global estimates of river flow wave travel times and implications for low-latency satellite data. *Geophysical Research Letters* **45**, 7551-7560 (2018).
